# Cotton morphological traits tracking through spatiotemporal registration of terrestrial laser scanning time-series data

**DOI:** 10.3389/fpls.2024.1436120

**Published:** 2024-08-01

**Authors:** Javier Rodriguez-Sanchez, John L. Snider, Kyle Johnsen, Changying Li

**Affiliations:** ^1^ School of Electrical and Computer Engineering, University of Georgia, Athens, GA, United States; ^2^ Department of Crop and Soil Sciences, University of Georgia, Tifton, GA, United States; ^3^ Bio-Sensing, Automation and Intelligence Laboratory, Department of Agricultural and Biological Engineering, University of Florida, Gainesville, FL, United States

**Keywords:** terrestrial laser scanning, crop morphological traits tracking, time-series field phenotyping, Spatiotemporal registration, 4D imaging, LiDAR, remote sensing

## Abstract

Understanding the complex interactions between genotype-environment dynamics is fundamental for optimizing crop improvement. However, traditional phenotyping methods limit assessments to the end of the growing season, restricting continuous crop monitoring. To address this limitation, we developed a methodology for spatiotemporal registration of time-series 3D point cloud data, enabling field phenotyping over time for accurate crop growth tracking. Leveraging multi-scan terrestrial laser scanning (TLS), we captured high-resolution 3D LiDAR data in a cotton breeding field across various stages of the growing season to generate four-dimensional (4D) crop models, seamlessly integrating spatial and temporal dimensions. Our registration procedure involved an initial pairwise terrain-based matching for rough alignment, followed by a bird’s-eye view adjustment for fine registration. Point clouds collected throughout nine sessions across the growing season were successfully registered both spatially and temporally, with average registration errors of approximately 3 cm. We used the generated 4D models to monitor canopy height (CH) and volume (CV) for eleven cotton genotypes over two months. The consistent height reference established via our spatiotemporal registration process enabled precise estimations of CH (*R* ^2^ = 0.95, RMSE = 7.6 cm). Additionally, we analyzed the relationship between CV and the interception of photosynthetically active radiation (IPAR*_f_*), finding that it followed a curve with exponential saturation, consistent with theoretical models, with a standard error of regression (SER) of 11%. In addition, we compared mathematical models from the Richards family of sigmoid curves for crop growth modeling, finding that the logistic model effectively captured CH and CV evolution, aiding in identifying significant genotype differences. Our novel TLS-based digital phenotyping methodology enhances precision and efficiency in field phenotyping over time, advancing plant phenomics and empowering efficient decision-making for crop improvement efforts.

## 1 Introduction

The increasing demand for agricultural products has emphasized the need for more efficient methods to accelerate crop productivity (Voss-Fels et al., 2019). Field-based plant phenotyping, key for evaluating plant traits in distinct environmental conditions, has become integral in crop breeding (Großkinsky et al., 2015). However, conventional techniques are laborious and time-consuming, often restricting assessments of traits to the end of the growing season and creating bottlenecks in breeding programs (Furbank and Tester, 2011). Recent advancements in remote and proximal sensing have increased the throughput and precision of field-based phenotyping (Singh et al., 2016). Nevertheless, persistent challenges in data processing and automation impede further progress in plant phenomics (Chawade et al., 2019; Kim, 2020). Therefore, novel strategies are imperative to streamline procedures and enable enhanced trait monitoring for crop growth tracking.

Integrating temporal data analysis into phenotyping pipelines can unveil plant development patterns and cyclic phenomena in growth. Continuous monitoring of plant growth is key for understanding plant behavior and responses to external factors (Miao et al., 2020). Recent studies have demonstrated the potential of continuous plant growth monitoring to identify specific genotypes contributing to particular traits (Xavier et al., 2017; Wang et al., 2018; Zhou et al., 2020; Li et al., 2022). However, the variability in crop growth within fields makes it challenging to establish standardized and consistent monitoring systems, hindering uniform trait tracking over time for large-scale fields (Tao et al., 2022). Exploring innovative and adaptable solutions for in-field trait assessment is key to overcoming these challenges and improving field phenotyping efficiency.

Advancements in three-dimensional (3D) imaging have expanded the application of plant modeling for the purpose of predicting crop traits over time. Detailed plant 3D models obtained from imaging data through photogrammetry methods (Paproki et al., 2012; Gelard et al., 2018; Hui et al., 2018), structured light scanning (Li et al., 2013), and active laser triangulation (Paulus et al., 2014) have been applied to track individual organs and monitor canopy growth of potted plants. However, these methods have proved labor-intensive and may lack scalability for larger field experiments. At the field scale, photogrammetry methods have also been proposed to gather 3D information from the crop over time using 2D imagery from ground (Carlone et al., 2015; Dong et al., 2017) or aerial systems (Chebrolu et al., 2018; Malambo et al., 2018). Despite their potential for large field coverage, these methods face constraints in capturing complex canopies due to RGB sensor limitations in handling occlusions, repetitive patterns, and changing light conditions.

In recent years, advanced methods using light detection and ranging (LiDAR) technologies for crop phenotyping have gained prominence. LiDAR scanners can mitigate some of the limitations associated with traditional 2D image sensors (Lin, 2015). Notably, employing a linear LiDAR scanning approach, mobile laser scanning (MLS) techniques have found application in field crop monitoring using moving ground vehicles (Jiménez-Berni et al., 2018; Sun et al., 2018) or specialized rail-based phenotyping platforms (Li et al., 2023). However, these methodologies involve analyzing crops from a fixed top-to-bottom orientation, potentially limiting the capture of lower canopy layers in dense crops with complex architectures, as certain parts of the crop may be shadowed or obscured from the sensor’s perspective.

Terrestrial laser scanning (TLS) techniques based on stand-alone 3D LiDAR scanners offer reliable solutions for overcoming occlusion issues and simplifying field-based crop phenotyping. Those scanners have demonstrated effectiveness in monitoring seasonal crop changes in small-scale breeding fields (Hosoi and Omasa, 2009, 2012) as well as daily canopy alterations in both individual plants (Herrero-Huerta et al., 2018) and large field trials (Jin et al., 2021a). Their consistent performance under various lighting conditions and superior depth-sensing capabilities make them particularly well-suited for precise crop canopy measurements in field-based plant monitoring (Madec et al., 2017; Jin et al., 2021b). However, the adoption of TLS techniques for plant phenotyping may be hindered by the absence of standardized and automated data processing methodologies (Medic et al., 2023), making continuous monitoring challenging. Simplifying LiDAR data processing and extraction of information can enhance plant trait analysis over time and promote broader usage in field phenotyping.

To ensure consistent crop growth modeling and plant traits monitoring using TLS, a fundamental step is to precisely associate spatial information from data collected over time into the same geospatial context. However, the complex transformations that crops undergo during the growing season complicate the registration of point clouds collected at different time points. The most straightforward technique for TLS-based crop growth tracking involves scanning the field from a fixed location at different points in time (Eitel et al., 2016). This single-position scanning approach introduces challenges such as laser shadows obscuring parts of the crop and variations in point density, particularly for plants closer to the laser scanner, that can impact subsequent data analysis (Malambo et al., 2019). An alternative approach involves scanning the field from multiple locations (e.g., multi-scan TLS). A study successfully employed this method to track wheat height over the growing season (Guo et al., 2019). Despite its advantages in reducing occlusions, this approach relied on precise geolocation of point cloud data, requiring accurate positioning systems that increase costs and processing time for data analysis (Crommelinck and Höfle, 2016). Another proposed method involves using a motorized gantry-type phenotyping platform to mount the LiDAR scanner and collect TLS data (Jin et al., 2021a). While effective for accurate trait monitoring, this approach requires a dedicated structure and highly accurate position sensors for real-time scanner location, introducing complexities to the phenotyping process.

To further enhance the precision and consistency of TLS-based time-series field phenotyping, the alignment of point clouds collected over time into a common coordinate system—4D registration— becomes fundamental. Traditionally, registering point clouds for crop growth monitoring has relied on the use of registration targets. This approach involves installing registration targets throughout the field to assist in aligning successive point clouds and has proven effective in monitoring crop growth in large breeding fields (Tilly et al., 2014; Friedli et al., 2016), as well as individual plants evolution under field conditions (Su et al., 2019). However, it can be labor-intensive and error-prone. Deviations in the target placement between surveys can impact the temporal alignment accuracy, and hence the estimation of traits over time. Ensuring that the registration targets are placed consistently in the same exact location in agricultural fields, where machinery needs to operate or other experiments need to be executed, can be challenging. More adaptable approaches used crop distribution to improve the alignment of remotely sensed data (Chebrolu et al., 2018; Günder et al., 2022). Although these studies were limited to aerial imagery, the concept could enhance spatiotemporal alignment of TLS data, facilitating its application for field phenotyping.

Alternative methods for 4D registration of point clouds have also been implemented to accommodate changes in plant structures, albeit with limitations for large-scale fields. Recent studies have proposed non-rigid registration methods to align point clouds at the organ-level during the growing season (Chebrolu et al., 2020; Magistri et al., 2020; Chebrolu et al., 2021). In contrast to rigid-body transformations (Besl and McKay, 1992), which assume a fixed relationship between the point clouds, non-rigid registration allows for more flexible and adaptive alignment, enabling more accurate tracking of plant growth and structural changes over time. However, these approaches require highly-detailed plant models, which can hinder their practicality for field crops where segregating individual plants might not be feasible. The development of methodologies for spatiotemporal registration of large-scale LiDAR data can significantly broaden the application of these technologies in field phenotyping, enabling efficient crop growth modeling and trait monitoring over time for enhanced crop development.

In this study, we present a novel methodology for automating the registration of field-based time-series TLS point clouds, addressing critical challenges in data registration and processing to facilitate consistent crop phenotyping and growth tracking. Our primary technical contribution lies in the development of a two-phase TLS data registration approach, which exploits terrain morphology and crop row distribution to minimize alignment errors between point clouds collected over time. This innovative method reduces dependence on fixed registration targets and streamlines the data processing pipeline, offering breeders a robust solution to acquire accurate phenotypic traits from the same physical locations at different time points. From a crop science perspective, our work introduces an efficient approach to track the evolution of crop traits throughout the season, enhancing our understanding of cotton plant development and trait variations. The specific objectives of the study were to: (1) develop a robust methodology for precise spatiotemporal registration of field-acquired point clouds; (2) validate the effectiveness of the methodology in reducing point cloud alignment errors throughout the growing season; (3) investigate the utility of registered time-series TLS data for extracting key morphological traits and facilitating precise monitoring of cotton crop growth dynamics; and (4) assess the applicability of the developed methodology in informing crop modeling efforts, with a focus on its potential to deepen our understanding of cotton plant growth and genotype-specific trait variations.

## 2 Materials and methods

### 2.1 Experimental field

The field research site used in this study was located at the University of Georgia’s Iron Horse research farm in Greene County, GA, USA (33°43’01.6”N 83°18’1.8”W). The field contained 11 different genotypes, arranged in a randomized complete block design with 8 replications. These genotypes included conventional Upland cotton varieties from public breeding programs (Acala Maxxa, DES 56, Tamcot Sphinx, UA 48), exotic genotypes (T0018MDN, T0246BC3MDN, and MDN0101 (GH191)), commercial Upland cultivars (DG 3615, DP 1646, and ST5020), and a Pima cotton cultivar (DP 314). A more detailed description of the included cultivars can be found in (Kaur et al., 2023).

**Figure 1:**
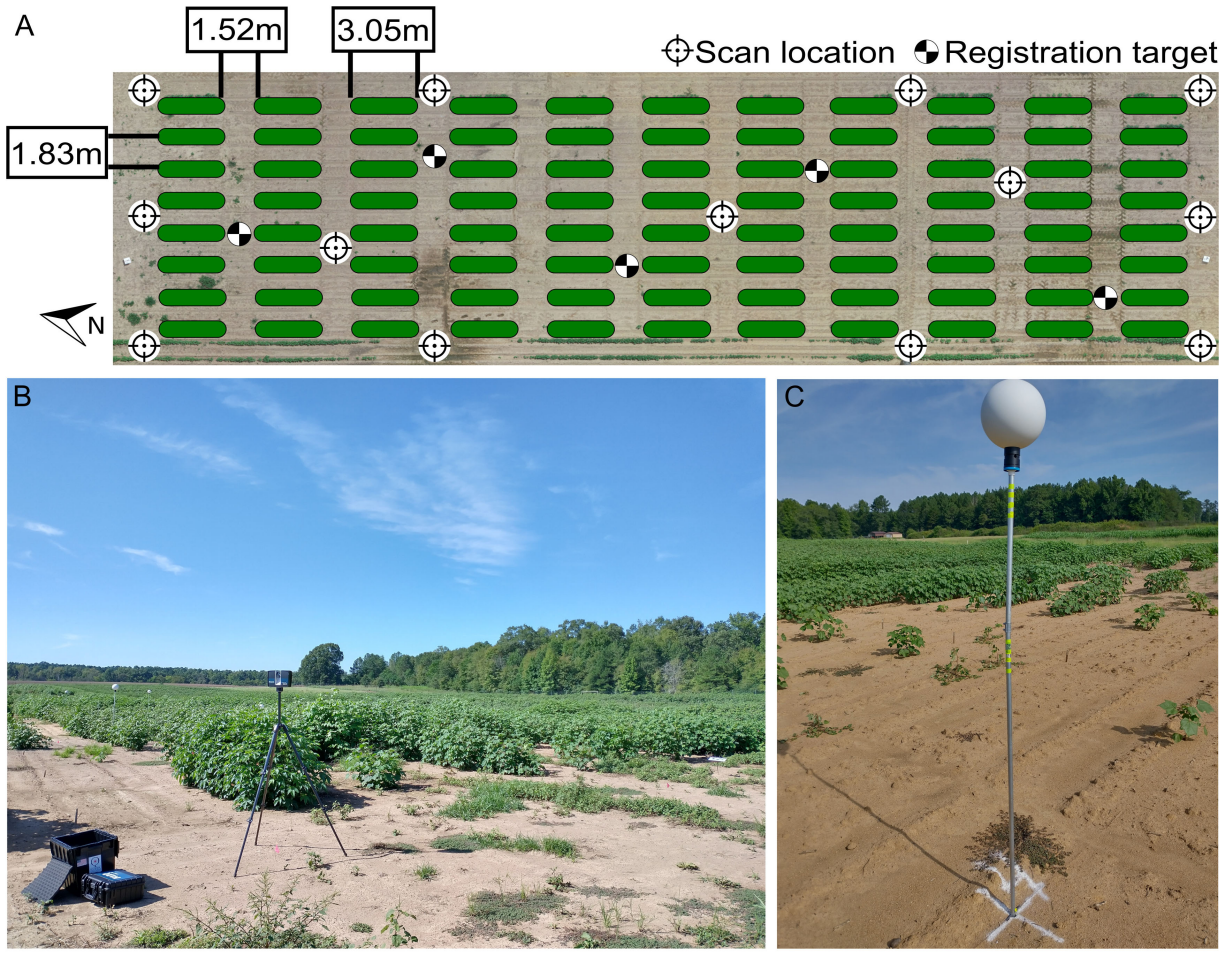
Experimental field layout. **(A)** Distribution of plots (green ovals), scan locations (circular target icons), and registration targets (checkered circles) for LiDAR-based phenotyping. **(B)** LiDAR scanner performing TLS scan at a scan location. **(C)** Registration target deployed in the field.

The field’s layout consisted of single-row plots, each measuring 3.05 meters in length, with an inter-row spacing of 1.83 meters (**Figure 1A**). A 1.52-meter wide bare soil alley separated each range of plots. The field was organized into 88 plots, distributed across 8 rows, with 11 plots per row, resulting in approximate dimensions of 52 meters in length and 14 meters in width. A total of 15 seeds were sowed in each plot on June 18, 2021 (planting date), and the final plant density in each plot varied based on germination and survival rates.

### 2.2 Field data collection

A FARO Focus 3D S70 laser scanner (FARO Technologies, Florida, US) was used for terrestrial laser scanning (TLS). To reduce occlusions and collect as much information from the crop as possible, a multi-scan methodology was adopted to scan the field. Between nine and thirteen scan locations, depending on the growth status of the crop, were used to ensure an adequate coverage of the crop during the different growth stages. The LiDAR was mounted on an elevating tripod (**Figure 1B**), whose height was adjusted *in situ* during each data collection session in accordance with crop height. At the beginning of the season, the height of the scanner was configured to 1.25 m. When the plants reached the canopy closure stage, the scanner height was adjusted to 1.8 m. The quality parameter for the LiDAR scanner was set to 2x, while the angular resolution was set to 1/2 (angular step of 0.18°) for both the vertical and horizontal angles, which is equivalent to a point distance of 3.05 mm over 10 m. The collected point clouds were colorized using information gathered from the color camera integrated in the LiDAR scanner. Additionally, global positioning system (GPS) and inclinometer information from the scanner’s internal sensors were also saved during the scan. For each scan location, raw point cloud data were stored in an SD card as FARO Laser Scan (.FLS) files for further processing.

Nine data collection sessions were conducted to monitor canopy development from crop establishment to canopy closure. The LiDAR scanner was used to survey the cotton field approximately at 35, 42, 49, 56, 62, 70, 77, 84, and 98 days after planting (DAP). During each session, five spherical targets with a diameter of 200 mm (Koppa Target Spheres, California, USA) were strategically placed throughout the field to facilitate the spatial co-registration of point clouds from different scan locations into a common coordinate system. These targets were mounted on aluminum rods with heights ranging from 1.4 m to 2 m (**Figure 1C**). The spherical targets were deployed before each TLS survey session. A mallet was used to drive the aluminum rods into the ground, ensuring each one of them went in straight and deep enough to provide stability during the survey. Since permanent structures in the field were not feasible due to ongoing experiments, the aluminum rods were taken down and stored between data collection sessions. Therefore, the position of the spheres was not necessarily consistent across different data collection sessions.

Manual measurements of key plant traits were conducted directly in the field at three different stages of crop development and served as ground truth data for validation purposes. The first session took place immediately after the onset of the blooming stage, approximately at 60 DAP. The second ground truth session occurred during the peak blooming period, at around 80 DAP. The final session was performed at the canopy closure stage, 90 just before the first open cotton bolls became visible. The field measurements included the height of the canopy and the quantity of light intercepted by the canopy, which is directly proportional to crop growth (Baker and Meyer, 1966) and plays a key role in modeling crop evolution and yield (Loomis and Williams, 1969). Canopy height (CH) was determined by measuring the distance between the ground and the top of the canopy (the plant terminal) using a measuring tape. Two to five different plants within each plot were measured following established methods commonly used in cotton breeding. The final CH value was calculated by averaging these measurements. Light interception was estimated by the fraction of photosynthetically active radiation (PAR) intercepted by the canopy (IPAR*_f_*), using Equation 1. Two IPAR*_f_* readings were taken per plot and then averaged. Light interception measurements were taken using an AccuPAR LP-80 ceptometer (METER Environment, Pullman, WA) under cloudless conditions between 1100 and 1300 h. Both the below-canopy photosynthetically active radiation (PAR*_below_*) and the above-canopy irradiance (PAR*_above_*) were measured simultaneously.


(1) $$IPAR_{f}=\frac{PAR_{above}-PAR_{below}}{PAR_{above}}$$

### 2.3 Data processing pipeline for spatiotemporal alignment

**Figure 2:**
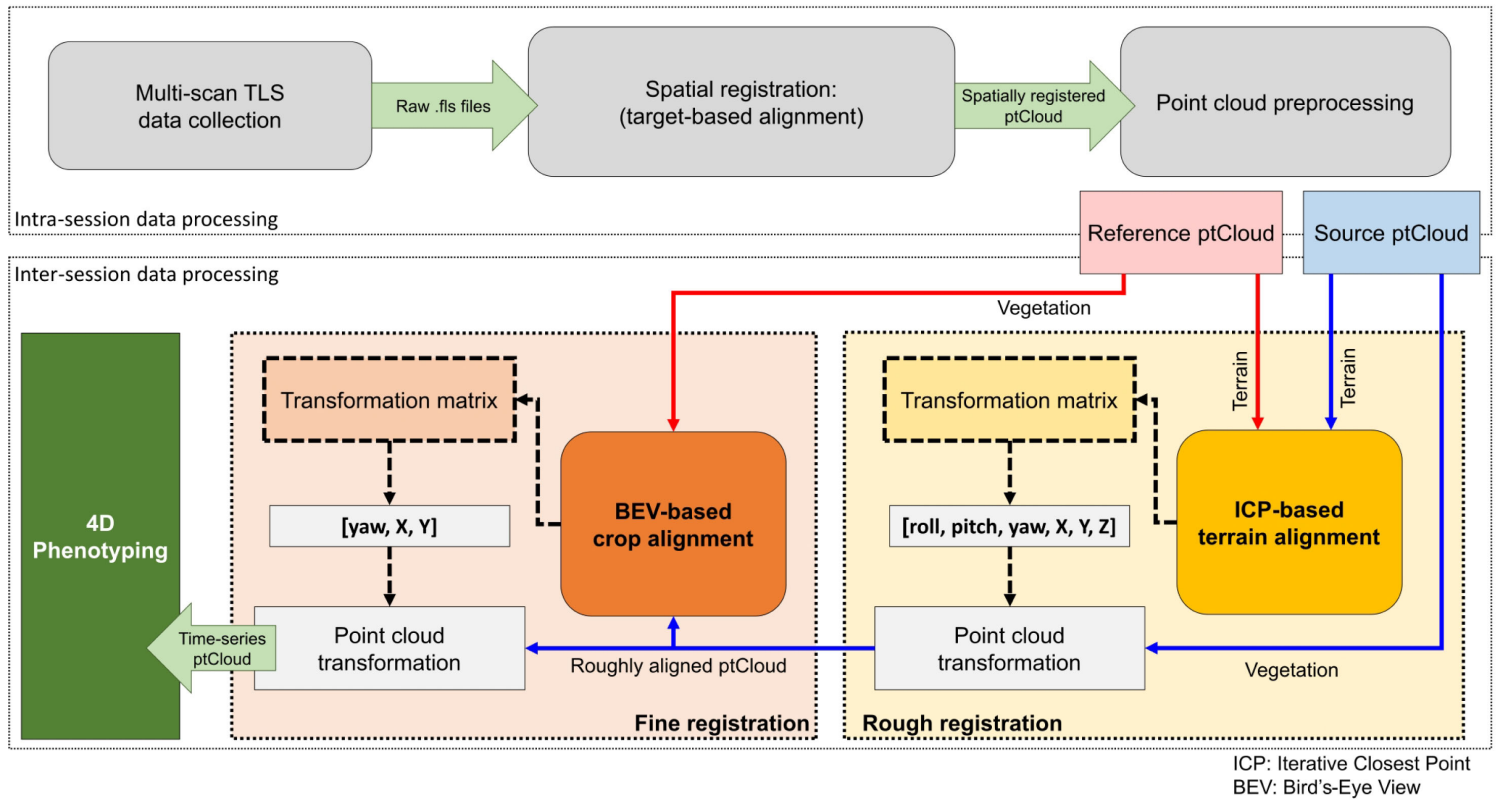
Overview of the proposed methodology for LiDAR-based 4D in-field phenotyping.

Our TLS-based 4D field phenotyping methodology involved a sequential series of data processing steps (**Figure 2**). Initially, intra-session data processing helped co-register multi-scan point clouds into a common coordinate system and prepared the data collected in each data collection for subsequent analysis. Inter-session processing aligned consecutive point clouds in both space and time, facilitating the extraction of crop traits to analyze growth trends over the season.

The data processing pipeline was designed to run on Windows systems, using only CPU resources without requiring specialized GPUs. All experiments were conducted on a desktop computer equipped with an 8-Core Intel(R) Core(TM) i7–9700K CPU running at 3.60 GHz and 64 GB of RAM.

#### 2.3.1 Intra-session data processing

After each data collection session, point clouds from multiple scan locations were co-registered using SCENE software (FARO Technologies, Florida, US), version 2019.2. The raw. FLS files were imported into SCENE and preprocessed using the *‘Edge artifact’* filter. Point clouds were automatically registered using the *‘Target Based’* method. Registration results were validated through target-based and mean point error statistics. Then, SCENE’s *‘Clipping box’* tool was used to isolate and extract 3D points within designated field boundaries. Points within the clipping box were saved in the LASer (.LAS) file format.

Co-registered point clouds underwent denoising using a statistical outlier removal (SOR) filter with parameters *N* = 20 and ±2.5 standard deviations as outliers boundaries. Then, a subsampling step based on a 5 mm point-to-point distance threshold reduced the point clouds size while maintaining spatial information, reducing computational demands. After subsampling, point cloud height was normalized with respect to a local digital terrain model (DTM) ensured consistency in elevation data across the field. For a more detailed description, please refer to the **Supplementary Materials**.

#### 2.3.2 Inter-session data processing

The terrain’s elevation profiles and slopes are generally stable in the short term, and the arrangement of crop rows remains consistent despite canopy growth. Leveraging this stability, we introduced a two-phase alignment process for temporally registering successive point clouds: one as the fixed reference and the other as the source undergoing alignment.

In the initial phase, we conducted a preliminary alignment using the reconstructed terrain models to establish a rough correspondence between pairs of point clouds. We used the iterative closest point (ICP) pairwise matching algorithm (Besl and McKay, 1992) to align the DTM points. The ICP algorithm seeks to find the best-fitting transformation that minimizes the distance between points in two point clouds, in our case the terrain points. This iterative process continues until a satisfactory alignment with minimum root mean squared deviation (RMS) is achieved. The resulting transformation matrix, including full rotation and translation components (i.e., roll, pitch, yaw, X, Y, and Z), was applied to the source point cloud, providing an initial spatial alignment.

**Figure 3:**
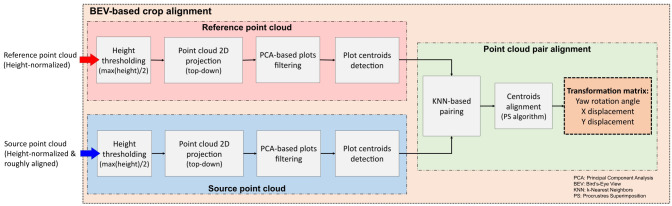
Bird’s-eye view alignment steps for multi-temporal point cloud pairs registration.

To further refine the alignment and minimize errors, we implemented a second phase based on bird’s-eye view (BEV) alignment (**Figure 3**). This approach used the distribution of crop rows and plots, automatically identified from the point clouds, to match the orientation and position of both point clouds from a top-down perspective. This refinement step ensured greater accuracy in aligning the point clouds collected over time.

The BEV alignment process started with the identification of plots centroids in both point clouds. A height threshold, set at half the maximum height value within each plot, was applied to separate points belonging to the upper canopy section. After thresholding, the canopy points were projected onto the X-Y plane, resembling a 2D top-down orthographic view of the field. Using contour detection, points corresponding to each plot were clustered and filtered using Principal Components Analysis (PCA) to eliminate incomplete plots or noisy point clusters. We leveraged the typical growth pattern observed in field crops planted in plots, where the length along the row axis generally exceeds the width. Any clusters deviating from this expected growth pattern were identified as noise and excluded from subsequent processing stages. The pixel coordinates corresponding to the centroid of each validated cluster were retained for further analysis.

The detected centroids served to improve the alignment between the point clouds. However, certain plots may not appear in the 2D projection in the XY plane during specific growth stages. To address this issue, a k-Nearest Neighbor (KNN) algorithm (Fix and Hodges, 1951) identified pairs of points common to both point clouds, excluding any missing centroids from the BEV alignment process. The rigid transformation between the source and reference point clouds was formulated as a Procrustes superimposition (PS) problem (Rohlf and Slice, 1990). This method involves determining the transformation needed to optimally align two sets of points, effectively overlaying one onto the other. Using a custom implementation of Sneath’s method (Sneath, 1967) (**Algorithm 1**), we calculated the translation and rotation required to align the centroids from the source point cloud with those from the reference point cloud.

Given a reference set *R* of *m* pairs of coordinates (x, y) representing the plot centroids detected in the reference point cloud, and a source set *S* of *n* pairs of coordinates (x, y) representing the plot centroids detected in the source point cloud, the PS algorithm returns the optimal translation and rotation angle that minimize the sum of the squared distances between corresponding points. First, *k* pairs of points

Algorithm 1 **Procrustes superimposition algorithm for plot centroids alignment.**

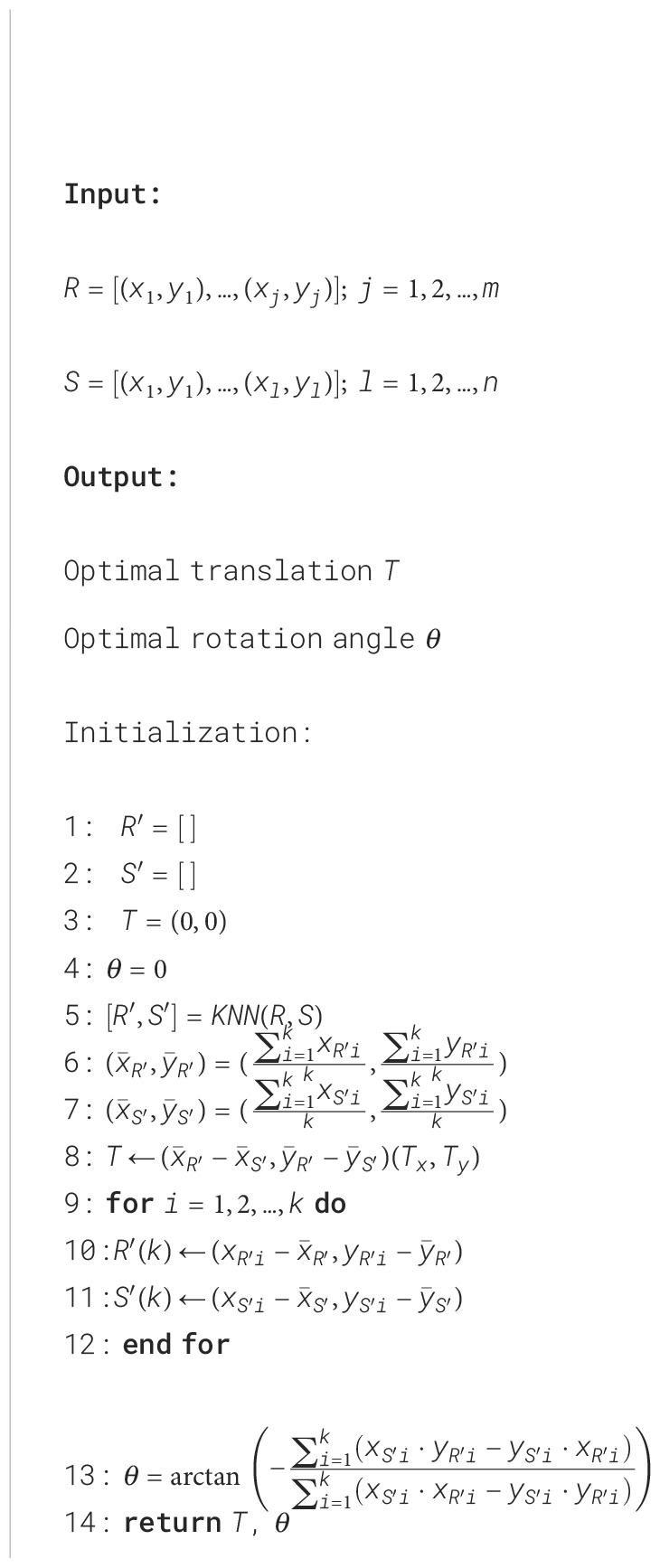

common to both point clouds were identified using the KNN algorithm (**Algorithm 1**, line 5). Then, the center of gravity (i.e., mean or average point) for these matched points was computed for each point cloud (**Algorithm 1**, lines 6 and 7). The distance between centers of gravity coordinates in the X and Y axes was used to compute the components of the optimal translation $\left(T_{x},T_{y}\right)$ (**Algorithm 1**, line 8). Then, both sets of points were translated to the origin (**Algorithm 1**, lines 9 to 12). Finally, the rotation angle was calculate using the sums of the cross products between x and y components for both sets to minimize the distances between corresponding points (**Algorithm 1**, line 13).

After fine registration, all point clouds were aligned under a common coordinate system. It is important to note that the initial normalization conducted in the previous section was performed locally for each data collection session. Discrepancies in DEM quality across different growth stages may introduce height errors between sessions, potentially impacting crop growth analysis. To mitigate these errors and minimize bias in trait estimation, height values for every data collection were renormalized using a common reference plane across all collected point clouds. The terrain mesh derived from the reference point cloud served as the global reference ground level (Z = 0 meters) for all subsequent point clouds. This approach ensured that traits measured relative to each plot’s unique characteristics had a consistent reference, enabling an unbiased assessment of crop growth over time.

#### 2.3.3 Performance analysis for spatiotemporal registration

To evaluate our methodology’s effectiveness in aligning point clouds over time, we needed accurately aligned reference models for each data collection session. We achieved this by manually aligning each point cloud dataset with the baseline reference, which was the initial point cloud collected at 35 DAP. Working in pairs and using the CloudCompare tool *‘Align (point pairs picking)’*, we selected 12 key points common to both point clouds. We then refined the alignment using the *‘Translate/Rotate’* tool until we achieved a root mean square (RMS) error of approximately 3 mm. The resulting transformation matrix served as the basis for comparing the temporal registration performance of our method.

To measure the performance of our registration process, we compared each point cloud spatiotemporally registered by our data processing pipeline with its manually aligned counterpart from each data collection session. The Hausdorff distance (Rote, 1991) was considered as performance metric to measure the dissimilarity between both point sets. Given two different point sets $PC_{A}=a_{1},a_{2},\ldots ,a_{n}$ and $PC_{B}=b_{1},b_{2},\ldots ,b_{n}$ , the Hausdorff distance from $PC_{A}$ to $PC_{B}$ can be computed using Equation 2:


(2) $$d_{H}\left(PC_{A},PC_{B}\right)=\underset{a\in PC_{A}}{\max}\underset{b\in PC_{B}}{\min}(d\left(a,b\right))$$

where *a* and *b* are points belonging to the point clouds $PC_{A}$ and $PC_{B}$ respectively, and d(a,b) is the Euclidean distance between *a* and *b*.

### 2.4 Crop traits estimation

#### 2.4.1 Individual-plot point clouds preparation

To isolate individual plots within the registered point clouds, we initially generated a polygon grid covering the entire field area and saved it using the ESRI shapefile (.shp) spatial data format. A region of interest (ROI) was manually defined for the first plot, then replicated and uniformly spaced to create a grid pattern aligning with crop rows and plots. The shapefile grid was used as the spatial guide to segment individual plots from each data collection session. As the point clouds were spatially and temporally co-registered with the reference point cloud from the first session, this grid generation was a one-time task applied throughout the growing season

#### 2.4.2 Plot-level traits extraction

After segmenting individual plots, key morphological traits, including canopy height (CH) and canopy volume (CV), were extracted using the *‘laspy’* and *OpenCV* libraries in Python. In addition, we evaluated the use of CV estimations as a proxy for estimating light interception under field conditions, which has been identified as a key input for process-based growth and yield models in cotton (Ermanis et al., 2020; Pokhrel et al., 2023). This indirect approach for estimating light interception has been explored previously in almond orchards (Zhang et al., 2021).

To estimate CH at the individual plot level, we analyzed normalized height values within each plot point cloud. We explored two percentile values, 95*^th^* (CH95) and 99*^th^* (CH99), as well as the maximum height (CHmax) derived from the histogram of Z-coordinates. We also compared CH estimations post-4D registration and those from individual point clouds before temporal registration.

For CV estimation, we initially calculated the per-plot projected canopy area (CA). Vegetation points were differentiated from terrain points based on a 5 cm threshold for Z-values. The identified vegetation points were then projected onto the XY plane, generating a 2D binary image mask for each plot. CA was determined by counting the number of pixels per unit area within the projected vegetation points. Subsequently, CV was estimated by multiplying the projected area by the corresponding CH values using Equation 3:


(3) $$CV_{projArea}=CA\cdot CH=numPixels\cdot \frac{shapeSize_{m}}{shapeSize_{px}}\cdot CH$$

where $shapeSize_{m}$ represents the shapefile area for the plot in m^2^, and $shapeSize_{px}$ represents the area in pixels.

#### 2.4.3 Crop trait estimation performance metrics

The overall performance of our methodology at estimating crop traits for field phenotyping was assessed by comparing trait values estimated from TLS data with ground truth values. The selection of the optimal height percentile for CH estimations was based in three metrics: the coefficient of determination (*R* ^2^), the root mean squared error (RMSE), and the mean absolute percentage error (MAPE), as defined in Equations 4–6, respectively. Higher values of *R* ^2^ approaching 1 and lower RMSE and MAPE values indicated more accurate estimations.


(4) $$R^{2}=1-\frac{\sum_{i=1}^{n}{\left(y_{i}-{\hat{y}}_{i}\right)}^{2}}{\sum_{i=1}^{n}{\left(y_{i}-\bar{y}\right)}^{2}}$$

(5) $$RMSE=\sqrt{\frac{1}{n}\cdot \sum_{i=1}^{n}{\left(y_{i}-{\hat{y}}_{i}\right)}^{2}}$$

(6) $$MAPE\left(\%\right)=\frac{1}{n}\sum_{i=1}^{n}|\frac{y_{i}-{\hat{y}}_{i}}{y_{i}}|\cdot 100$$

where *n* is the total number of data points used for regression analysis, $y_{i}$ is the actual value of CH for the *i^th^* ground truth plot, ${\hat{y}}_{i}$ is the predicted CH value obtained from the LiDAR point cloud associated with the *i^th^* ground truth plot, and $\bar{y}$ is the mean CH value calculated from the total of ground truth measurements.

To assess the use of CV estimations as a proxy for estimating canopy light interception, a nonlinear regression analysis was performed. This analysis compared the estimated CV values with field ground truth measurements of IPAR*_f_*, using nonlinear least-squares fitting with the *‘curve fit’* function from the *‘scipy’* Python library. The standard error of regression (*SER*) was calculated using Equation 7 to evaluate the goodness of fit, accounting for the number of independent variables.


(7) $$SER=\sqrt{\frac{1}{n-\left(p+1\right)}\cdot \sum_{i=1}^{n}{\left(y_{i}-{\hat{y}}_{i}\right)}^{2}}$$

Here, $y_{i}$ and ${\hat{y}}_{i}$ are the actual and predicted values of IPAR*_f_* for the *i^th^* ground truth plot, respectively, *n* is the total number of data points used for nonlinear regression, and *p* is the number of coefficients in the model not counting the intercept.

#### 2.4.4 Growth modeling and parameters estimation

The growth cycle of cotton plants follows an S-shaped curve, with lower and upper bounds indicating the period of growth equilibrium (Snider et al., 2021). Among the mathematical models for growth modeling, the three most commonly used for asymptotic growth are the logistic model, the Gompertz model, and the Richards growth model (Tjørve and Tjørve, 2010) (**Supplementary Table S1**). These models capture plant growth trajectories with different levels of asymmetry and flexibility. We defined *W* as the trait value at a given time *t*, *A* as the maximum value for the trait (upper asymptote), *k* as the growth rate, and *T_i_* as the time at inflection when the maximum growth rate occurs.

The three sigmoid growth models were used to analyze the estimated trait data and reveal the underlying dynamics of CH and CV evolution over time. Nonlinear mixed-effect models (NLME) were employed to capture trait evolution, considering *time* and *genotype* as fixed effects, while *replicate* was treated as a random effect. For the model fitting process, we used the statistical computing and graphics software R (R Core Team, 2023), version 4.2.3, and the *‘nlme’* package (Pinheiro and Bates, 2000). For the sake of simplicity and to ensure convergence, we assumed that the growth rate parameter *k* remained constant and independent of genotype, without showing any random variability across replicates.

The likelihood ratio test (*‘anova.lme’*) from the *‘nlme’* package was used to identify the most suitable model based on the Akaike information criterion (AIC) (Akaike, 1992) and the Bayesian information criterion (BIC) (Schwarz, 1978) metrics. The model with the highest AIC and BIC support was fitted to the trait data, estimating growth curve parameters. A *post-hoc* analysis was conducted to test for mean differences in growth parameters by genotype using the *‘emmeans’* package (Lenth, 2023), with Bonferroni corrections for multiple comparisons. This comprehensive approach enabled us to discern significant variations in growth characteristics across different genotypes.

## 3 Results

### 3.1 Development of the 4D field model for validation

**Figure 4:**
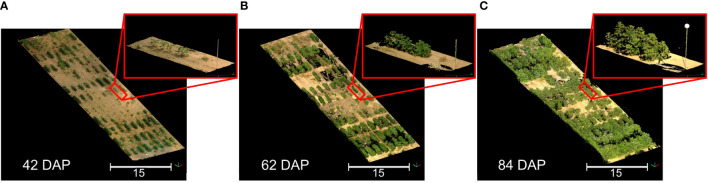
3D representation of the field at three different growth stages. **(A)** Data collected 42 days after planting (DAP); **(B)** Data collected 62 DAP; **(C)** Data collected 84 DAP. Point clouds were colorized using information from the sensor’s camera. Black areas represent spaces without 3D point information. Insets show a close-up of the same plot in each point cloud.

We successfully reconstructed detailed point clouds for each data collection session using multi-scan TLS approaches and automatic registration (**Figure 4**). The average mean point distance between matched target pairs was 1.6 mm, with a maximum error of 3.2 mm across all sessions. On the whole, the average mean scan point error remained consistently below 6 mm, peaking at 8.5 mm for data collected at 84 DAP. After point cloud preprocessing, the point cloud density was reduced by 8 to 18 times (**Supplementary Table S2**), significantly decreasing LAS file sizes to approximately 0.5 GB each and facilitating subsequent processing. This reduction also enabled simultaneous processing of all point clouds in a single CloudCompare session.

**Figure 5:**
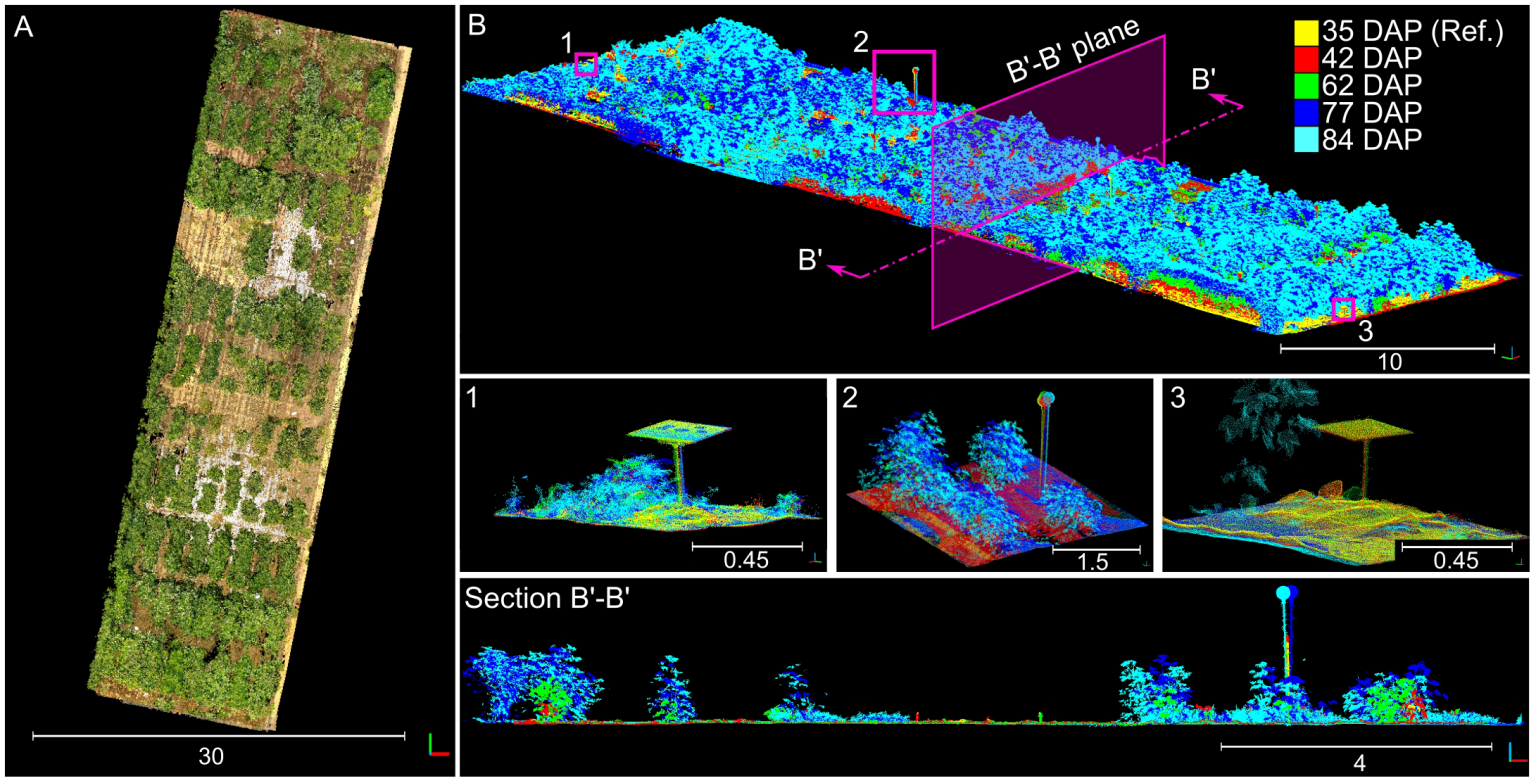
Manually aligned time-series point cloud used for benchmarking. **(A)** Overhead view of the reconstructed time-series point cloud; 3D points were colorized using information from the sensor’s camera. **(B)** Perspective view of the point cloud colorized by data collection session; numbered insets show close-up views for the distinctive objects enclosed in pink boxes. Section B’-B’ shows a slice of the point cloud taken from the direction indicated by the pink arrows. Different solid colors denote point clouds collected at different dates.

The presence of consistent and distinguishable 3D features across all collected point clouds allowed for the creation of an accurate 4D field model (**Figure 5**). This model integrated individually reconstructed 3D point clouds from each session, aligning them to the reference point cloud under a unified coordinate system. As a result, we obtained almost perfectly aligned point clouds for each session that served as a crucial reference for validating the accuracy of our registration process. By comparing the individual point clouds after alignment using our methodology to the reference model, we were able to assess the performance of our registration method.

### 3.2 Terrain-based registration of point clouds

**Figure 6:**
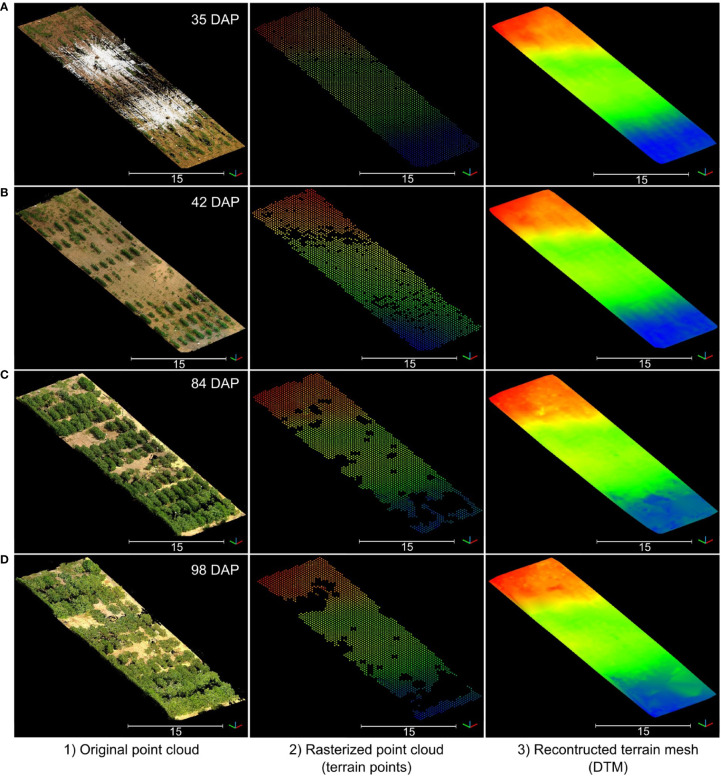
Reconstructed digital terrain model (DTM) for four data collection sessions during the vegetative growth stage. Rows **(A–D)** correspond to data collected at 35 days after planting (DAP), 42 DAP, 84 DAP, and 98 DAP, respectively. For each row, from left to right: (1) RGB point cloud; (2) Rasterized point cloud colorized by elevation; and (3) Reconstructed DTM colorized by elevation. Elevation is represented using a color map ranging from blue (the lowest point) to red (the highest point).

The rasterization of terrain points and the posterior meshing process allowed us to obtain consistent DTM models for each data collection session during the growing season (**Figure 6**). However, the reconstruction process tended to overestimate terrain elevation in areas with excessive vegetative growth and denser canopies. As the growing season progressed and plant canopies began to overlap with neighboring plots, the number of terrain points collected by the LiDAR scanner notably decreased, leading to data gaps in the rasterized point clouds (**Figures 6B–D**). These gaps presented a challenge in accurately modeling the terrain. Notably, when comparing the initial terrain model from the first data collection session (**Figure 6A**) to the final session (**Figure 6D**), disparities in elevation reached nearly 37 cm in regions with dense vegetation. Despite these challenges, our method consistently produced accurate terrain morphology results up to 84 DAP when all plots in the field effectively reached the canopy closure stage.

Leveraging internal sensors data gathered during LiDAR data collection allowed us to position consecutive point clouds in close proximity to each other and facilitated convergence of the ICP algorithm, minimizing its risk of getting trapped in local minima (**Supplementary Figure S2**). The resulting RMS values for ICP registration between the DTM for each data collection session and the reference DTM were consistently less than 10 mm.

### 3.3 Bird’s-eye view-based alignment for spatiotemporal registration refinement

**Figure 7:**
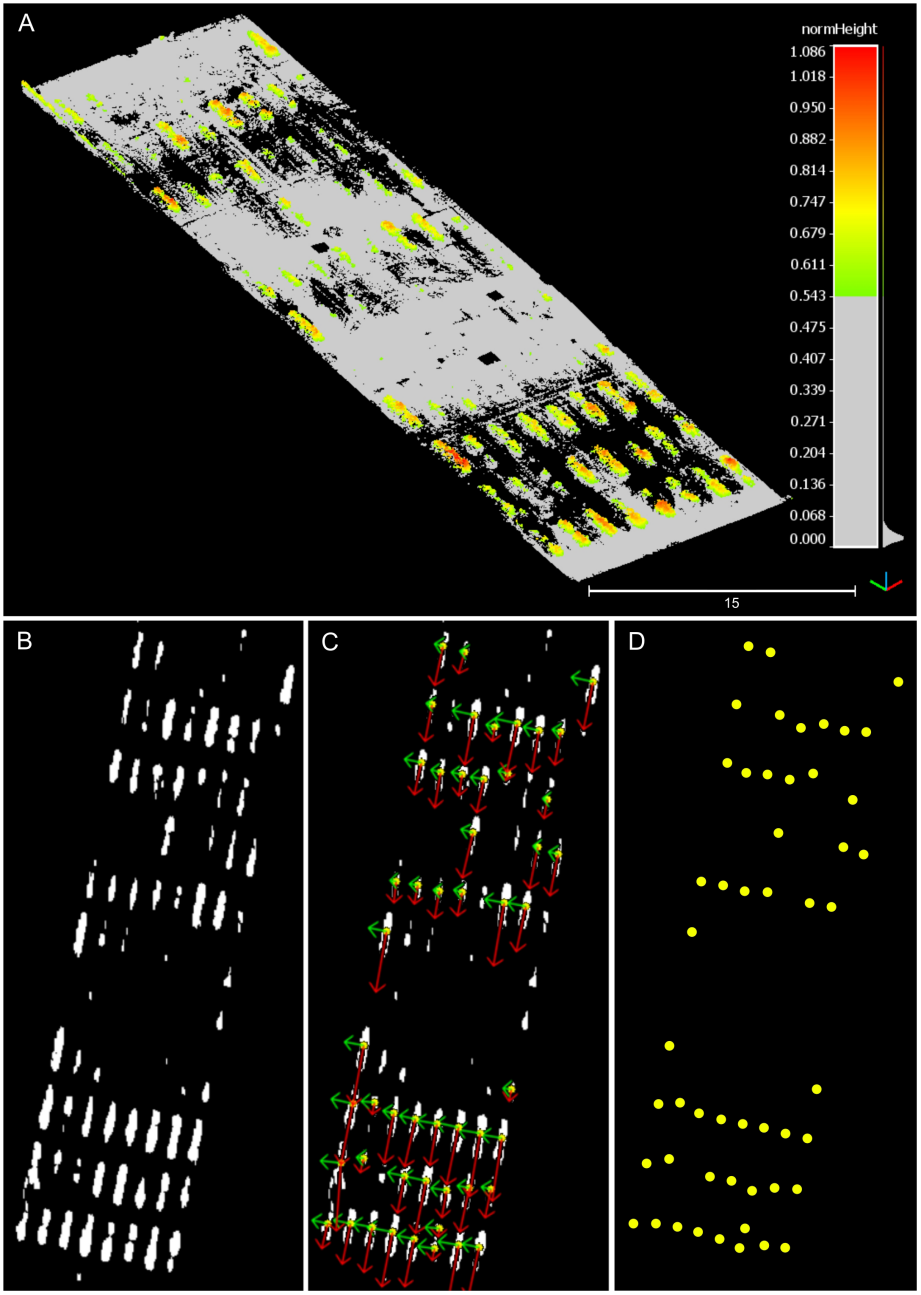
Partial results during the bird’s-eye view alignment process. The depicted point cloud data pertains to the data collection session conducted at 42 DAP. **(A)** Canopy points isolation after height thresholding. **(B)** Projection of canopy points onto the X-Y plane in 2D. **(C)** Identified pixel clusters and principal component analysis results. Red arrows depict the first principal component’s direction, while green arrows illustrate the second principal component’s direction. **(D)** Visual depiction of identified plot centroids.

The developed process enabled the isolation of prominent plots in the field, allowing for an accurate identification of their centroids (**Figure 7**). Through local height normalization and thresholding, only the upper section of the canopy was retained (**Figure 7A**), revealing the distinct pattern of crop rows by focusing solely on vegetation points (**Figure 7B**). Following plot clustering, PCA-based filtering reduced the potential impact of spurious points or poorly defined plots, such as noise, small plots, or tall weeds, thereby enhancing the consistency and robustness of plot centroid identification (**Figures 7C, D**). For a comprehensive visualization of the BEV alignment process for two point clouds, refer to **Supplementary Figure S3**.

**Figure 8:**
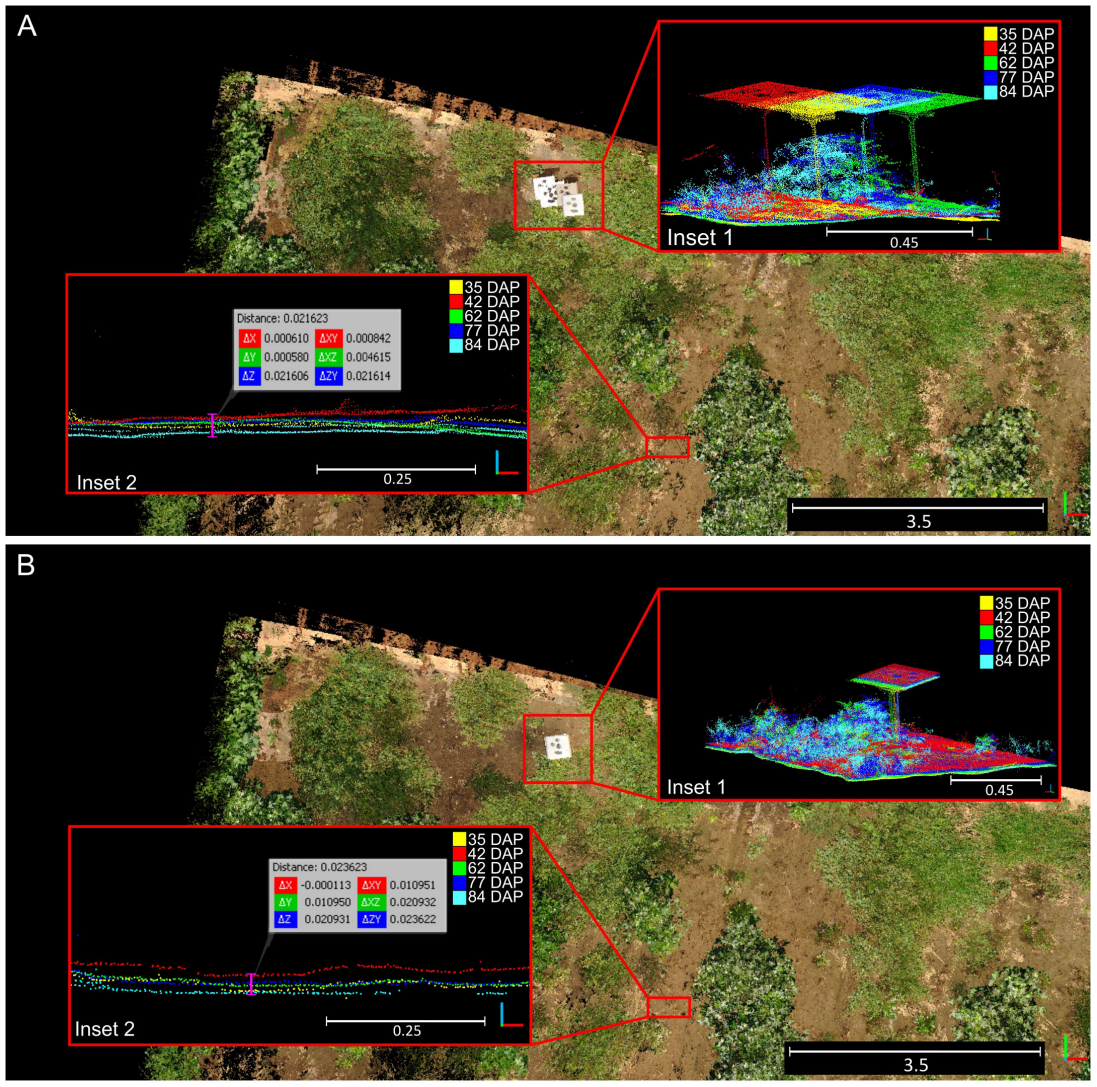
Qualitative results for the two-step registration process for point clouds collected over time. **(A)** Rough alignment results after the first alignment step based on digital terrain model matching. **(B)** Final alignment achieved after bird’s-eye view refinement. Insets (1) demonstrate generalized alignment errors; Insets (2) highlight errors in the Z direction at the terrain level. Different solid colors denote point clouds collected at different dates.

The BEV alignment step consistently enhanced the accuracy of point cloud registration for data collected during the vegetative crop growth stage (**Figure 8**). Initially, errors ranging between 10 cm and 35 cm with respect to the reference point cloud were observed after the first alignment step (**Figure 8A**, inset 1). However, after the second alignment step, errors for points above the terrain plane notably decreased to values around 2 to 3 centimeters (**Figure 8B**, inset 1). Examination of alignment results at terrain level revealed that registration errors in the Z direction remained relatively constant, increasing slightly from 2.2 cm for the initial DTM-based alignment step (**Figure 8A**, inset 2) to 2.3 cm for the refinement step based on BEV (**Figure 8B**, inset 2).

In general, the alignment of point clouds remained consistent throughout the growing season, with final alignment errors comparable to those achieved by manual alignment of the point cloud pairs. In the manually aligned point clouds, the distance between point clouds at the overall level ranged between 0.5 and 1 cm, while at the ground level, the distance in the Z axis between point clouds reached approximately 6 mm. To see a detailed visual comparison between the manually aligned point clouds and those registered using our methodology, refer to **Supplementary Figure S4**.

### 3.4 Quantitative analysis of errors for spatiotemporal registration

Our quantitative analysis confirmed significant reductions in the main distances between point clouds registered using our methodology and their manually aligned counterparts (**Table 1**). After the initial alignment based on DTM matching, the average Hausdorff distance between point clouds was approximately 31 cm, consistent with our previous qualitative analysis (**Figure 8**). With the second alignment step, the average Hausdorff distance was reduced to 5.5 cm, representing an 83% reduction from the initial alignment errors. However, despite this improvement, the alignment error for the final data collection session remained relatively high, reaching 13.5 cm even after the refinement step, in contrast to the approximately 3 cm observed for the other point clouds. This difference can be attributed to the limitations of the initial alignment based on the DTM in advanced growth stages. The denser and taller canopy obstructed the laser beam during the survey more frequently, resulting in limited terrain data for ICP registration. This contributed to larger initial errors that the refinement step, based on the BEV alignment approach, was not able to completely rectify.

**Table 1:** Spatiotemporal registration errors after each alignment step.

	Step 1 Terrain model-based alignment								Step 2 Bird’s-eye view-based alignment							
			$d_{H_{X}}$		$d_{H_{Y}}$		$d_{H_{Z}}$				$d_{H_{X}}$		$d_{H_{Y}}$		$d_{H_{Z}}$	
DAP	RMS↓ (std)	$d_{H}$	max	min	max	min	max	min	RMS↓ (std)	$d_{H}$	max	min	max	min	max	min
42	2.1 (1.9)	19.3	18.3	-16.9	16.1	-18.3	17.6	-15.8	1.2 (0.6)	3.2	3.0	-3.1	3.0	-3.1	3.2	-3.1
62	4.9 (6.5)	32.5	32.0	-32.3	31.0	-30.0	31.8	-29.4	0.5 (0.3)	2.4	2.1	-2.1	2.1	-2.1	2.1	-2.1
84	3.6 (3.6)	24.2	22.3	-24.0	21.8	-22.6	23.0	-22.4	0.8 (0.5)	3.5	3.2	-3.0	3.0	-3.1	3.2	-3.0
98	5.7 (5.7)	46.8	36.3	-46.1	43.5	-36.9	39.7	-40.1	2.5 (1.8)	13.5	11.7	-12.5	12.2	-11.4	12.3	-11.3

All values are given in centimeters.

**Figure 9:**
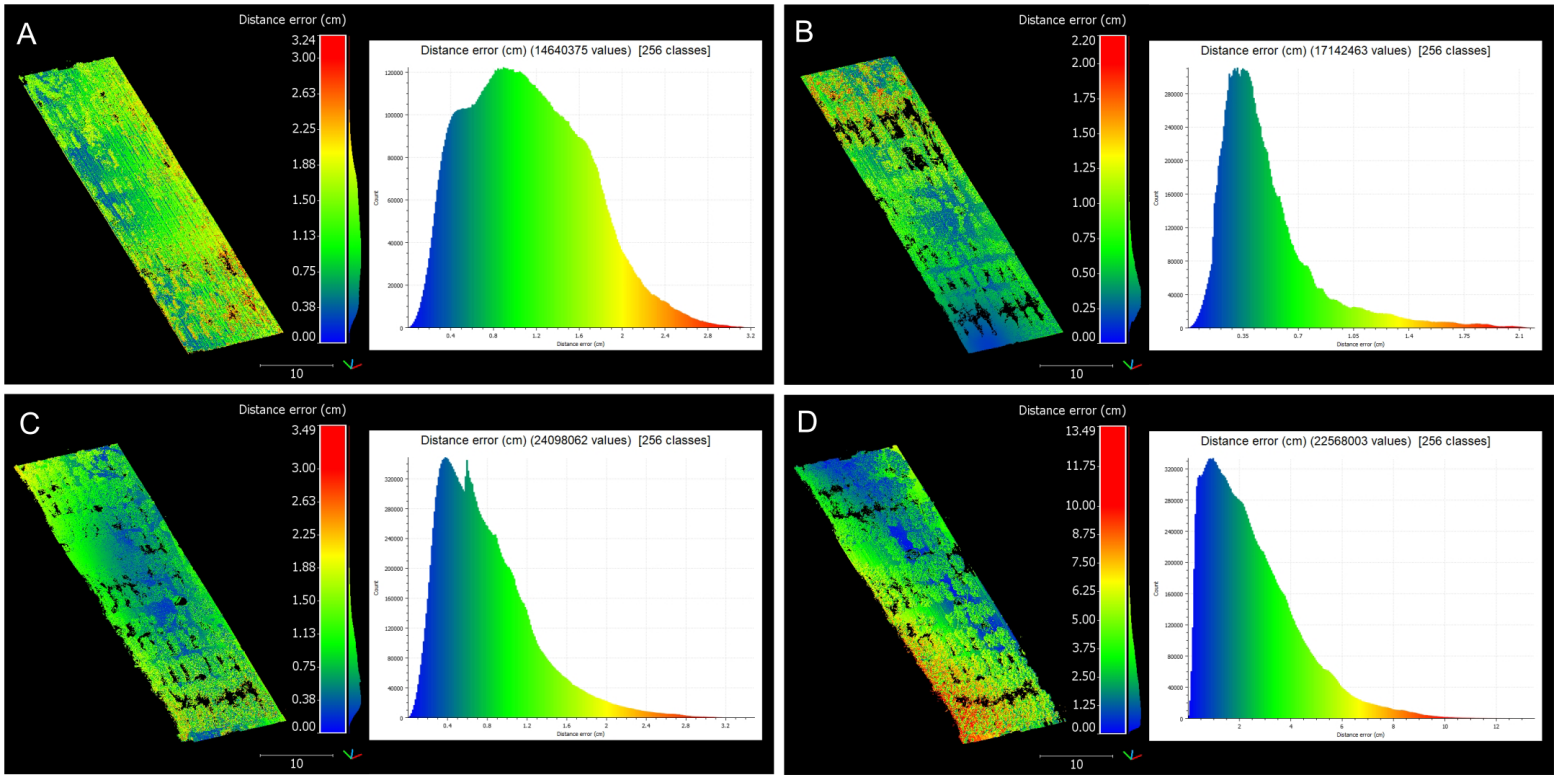
Distribution of registration errors across data collection sessions. Comprehensive analysis of registration errors showcasing both the spatial distribution of distance errors and their respective histograms. **(A)** 42 days after planting (DAP). **(B)** 62 DAP. **(C)** 84 DAP. **(D)** 98 DAP. The distribution of distance errors is visualized with a color gradient ranging from blue (low error) to red (high error).

A deeper analysis of registration errors revealed a clear trend: as the cotton canopy grew and became denser, the registration errors increased both in magnitude and variability (**Figure 9**). Early stages showed relatively low and uniform errors, while later stages exhibited higher errors due to the complexity of the mature canopy. This progression of errors from the early to maturity stages demonstrated the increasing difficulty in point cloud registration as the crop canopy developed, highlighting the importance of robust registration methodologies to handle increased canopy density and complexity.

At 42 DAP, during the early-stage canopy development with minimal plant overlap, distance errors peaked at 3.24 cm, with most errors being moderate. The majority of errors ranged between 0.38 cm and 1.13 cm, suggesting a low error spread. At 62 DAP, with increased canopy coverage and uniform growth, the maximum error reached 2.20 cm, with errors more uniformly distributed across the field compared to the earlier stage. Errors were primarily concentrated between 0.25 cm and 0.75 cm, reflecting improved registration accuracy during this stage. This improvement could be attributed to better performance of the BEV refinement step, where increased canopy density provided more information to identify plot centroids, enhancing alignment accuracy. Additionally, as the crop grew taller, interference from weeds diminished, aiding in more accurate plot identification.

By 84 DAP, when the crop already reached canopy closure stage, the maximum error increased to 3.49 cm, with higher variability in areas of dense canopy growth. Errors ranged more broadly, with a significant portion between 0.75 cm and 2.63 cm, highlighting the challenges of maintaining accuracy as the canopy became denser. This stage reflected the increasing complexity of the registration process as the canopy structure became more intricate and plots overlapped, posing significant challenges to maintaining low error rates. At 98 DAP, errors reached a maximum of 13.49 cm, demonstrating significant challenges due to dense and overlapping canopies. A wide distribution of errors, clustering between 2 cm and 10 cm, indicated substantial variability and the presence of outliers caused by canopy occlusion and overlapping. At this point, the refinement step was no able to reduce alignment errors and optimize registration.

### 3.5 Phenotypic traits estimation

Our spatiotemporal registration methodology significantly enhanced CH estimations compared to conventional TLS data analysis without temporal registration (**Supplementary Table S3**). All analyzed percentiles for CH estimation—95*^th^*, 99*^th^*, and maximum height—exhibited a strong relationship with actual measurements for both methods. However, after the two-step spatiotemporal registration process, CH estimations consistently demonstrated stronger correlations with ground truth measurements and reduced errors compared to estimating CH from individual point clouds without temporal co-registration.

Estimations of CH using the maximum height value consistently exhibited the strongest correlation with actual CH values, explaining almost 95% of the total variance. While CH95 and CH99 also demonstrated strong correlations with ground truth measurements, CHmax consistently outperformed them across various evaluation metrics. Notably, CHmax showed reduced average deviation from the actual values and lower errors compared to CH95 and CH99, with an RMSE below 8 cm and average deviation of about 5%. These results are in line with findings from previous LiDAR-based studies in cotton (Sun et al., 2017, 2021), indicating the reliability of CHmax for estimating canopy height in cotton crops from multi-scan TLS, particularly in the context of time-series data analysis. CHmax captures the tallest point in the canopy, akin to manual field measurements, making it less susceptible to variability in canopy structure compared to percentile-based estimations, especially after denoising the point clouds.

#### 3.5.1 Overall performance analysis

**Figure 10:**
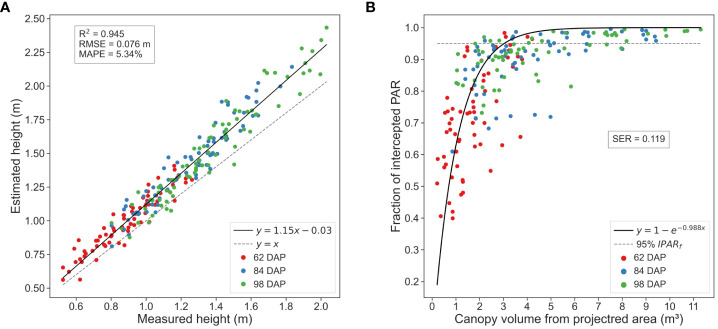
Phenotypic traits estimation results. **(A)** Canopy height estimations computed using the maximum height value versus ground truth measurements. **(B)** Canopy volume estimations versus ground truth measurements of fraction of intercepted photosynthetic active radiation (PAR).

Our methodology for processing and analyzing in-field time-series TLS data demonstrated robust performance in estimating key traits versus ground truth measurements (**Figure 10**). The regression analysis showed a strong agreement between estimated values and ground truth measurements for both CH and IPAR*_f_*. Despite the complex and evolving nature of the crop, our methodology allowed for the precise estimation of these traits, accurately capturing the dynamic changes in crop traits over time.

Regarding CH estimations, we observed a general slight overestimation of canopy height compared to the actual values, which became more pronounced as the crop matured (**Figure 10A**). The linear regression model displayed a slight deviation from the 1:1 line, a trend consistent across other percentiles, albeit with slight variations. For instance, analysis of the 95*^th^* percentile revealed a more pronounced tendency to overestimate canopy height during early growth stages, suggesting it may be less reliable for estimating in-field cotton plots height.

Analyzing the results for each data collection session individually, error values showed an increasing trend with crop development, leading to more dispersed CH estimations for larger canopies during later growth stages. RMSE values varied approximately 2 cm from the initial session to the last. At 62 DAP, the RMSE surpassed 6 cm, increasing to approximately 7 cm at 84 DAP and 8 cm at 98 DAP. This trend may be attributed to diverse canopy development of plants amongst plots or plant lodging during the season. Other potential causes of errors included underestimation in terrain elevation in regions with dense vegetative growth, as discussed previously (Section 3.2), or potential human error, given the challenges of measuring tall (reaching more than 2 m) and dense crops in the field. Nevertheless, examination of MAPE values revealed a consistent performance of our methodology, with only a 1.42% difference between the maximum and minimum MAPE values. Specifically, at 62 DAP, MAPE reached 6.17%, at 84 DAP it was 4.76%, and at 98 DAP, 5.11%. This suggests that, despite the observed increase in RMSE, our estimations remained relatively close to the actual values across different growth stages.

Upon examining the estimated CV in relation to field measurements of IPAR*_f_*, we observed a pattern of exponential saturation with a distinct asymptote as the crop canopy approached its maximum volume (**Figure 10B**). This pattern aligns with the classical Beer-Lambert’s law of attenuation of light through the canopy, offering a straightforward approach for predictive crop physiological traits estimation. Notably, the critical leaf area index at which 95% of radiation is intercepted was reached at canopies volumes between 3 and 4 *m* ^3^. The crop attained these volumes in a generalized manner between the first and second ground truth data collection dates, suggesting the likely canopy closure between 62 DAP and 84 DAP, a timeframe consistent with bibliography on cotton crop physiology (Snider et al., 2021). These findings provide valuable insights into the dynamic interplay between canopy structure and light interception efficiency, key for optimizing crop productivity.

#### 3.5.2 Validation of CH estimates over time

**Figure 11:**
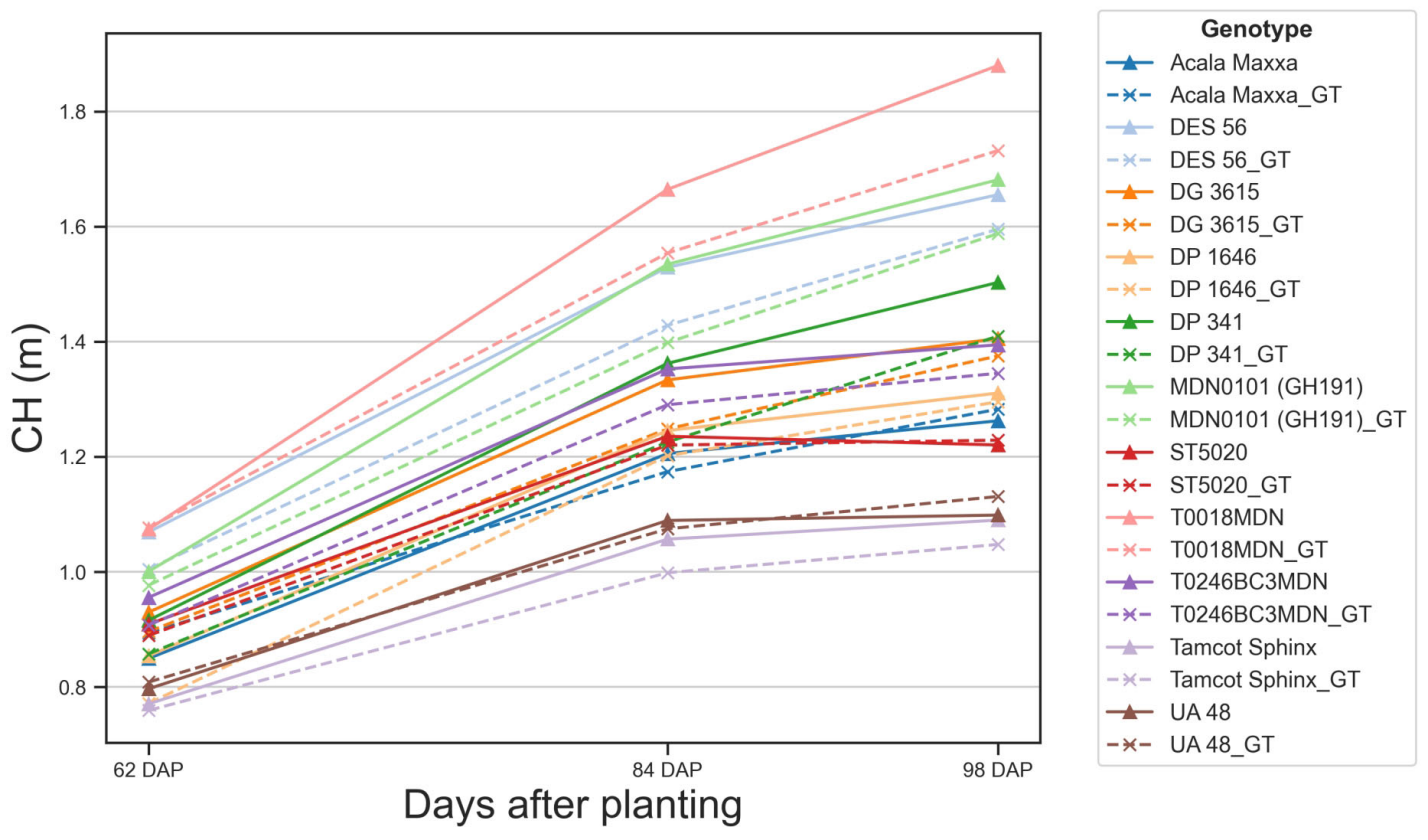
Temporal variation of predicted and observed canopy height. Solid lines represent canopy height (CH) values estimated using our methodology. Dashed lines represent ground truth (GT) CH values measured manually in the field. Each color and symbol combination represents a different genotype, with corresponding pairs (e.g., Acala Maxxa and Acala Maxxa_GT) for each genotype.

Our spatiotemporal registration of TLS data provided reliable estimations of CH over time (**Figure 11**). The comparison between TLS-based estimates and ground truth measurements indicated that the temporal variation of predicted and observed data was generally within 7 cm for most genotypes and data collection sessions. This agreement was particularly strong for canopies under 1.5 meters, where CH estimations closely matched actual values. However, for canopies exceeding 1.5 meters, our estimations tended to slightly overestimate CH, which is consistent with our previous regression analysis.

The proximity of our estimated values to actual measurements indicated the accuracy of our methodology. CH estimations generally followed trends of steady growth, maintaining close alignment with ground truth values. In several cases, such as UA 48 or ST5020, the estimated CH closely matches the ground truth, demonstrating the reliability of our scanning technique. However, we found some discrepancies between estimated and ground truth values for certain genotypes, such as T0018MDN or MDN0101, highlighting areas for potential refinement in our scanning process. These discrepancies can be attributed to two main factors. Firstly, these two genotypes showed the highest growth rate, and as the canopies grew taller and denser, measuring their height in the field became more challenging and error-prone, introducing noisy points that could mask the performance of our methodology. Secondly, the increased complexity of the canopy structure at later growth stages might have affected the accuracy of the TLS data registration, leading to slight overestimations in CH.

Our methodology effectively captured the variations in CH among different genotypes over time. Some genotypes exhibited considerable growth, and our methodology tracked this evolution relatively accurately. At 62 DAP, CH among genotypes showed minimal variation, mostly clustered between 0.8 and 1.0 meters, and our estimation errors were below 5 cm. However, as the crop matured, our CH estimates began to slightly diverge from the actual values. By 98 DAP, there was greater diversity in CH, ranging from nearly 1.8 meters for some genotypes to around 1.2 meters for others. At this stage, TLS estimates tended to be higher than the actual measurements, with estimation errors reaching approximately 20 cm for certain genotypes. These observations were consistent when analyzed at the individual plot level (**Supplementary Figure S5**). For genotypes such as DES 56 or DG 3615, average estimation errors remained under 7 cm. However, in plots of genotypes like T0018MDN, errors approached 25 cm at later DAP, indicating challenges in accurately measuring CH as canopies became taller and denser.

### 3.6 Analysis of phenotypic traits over time

**Figure 12:**
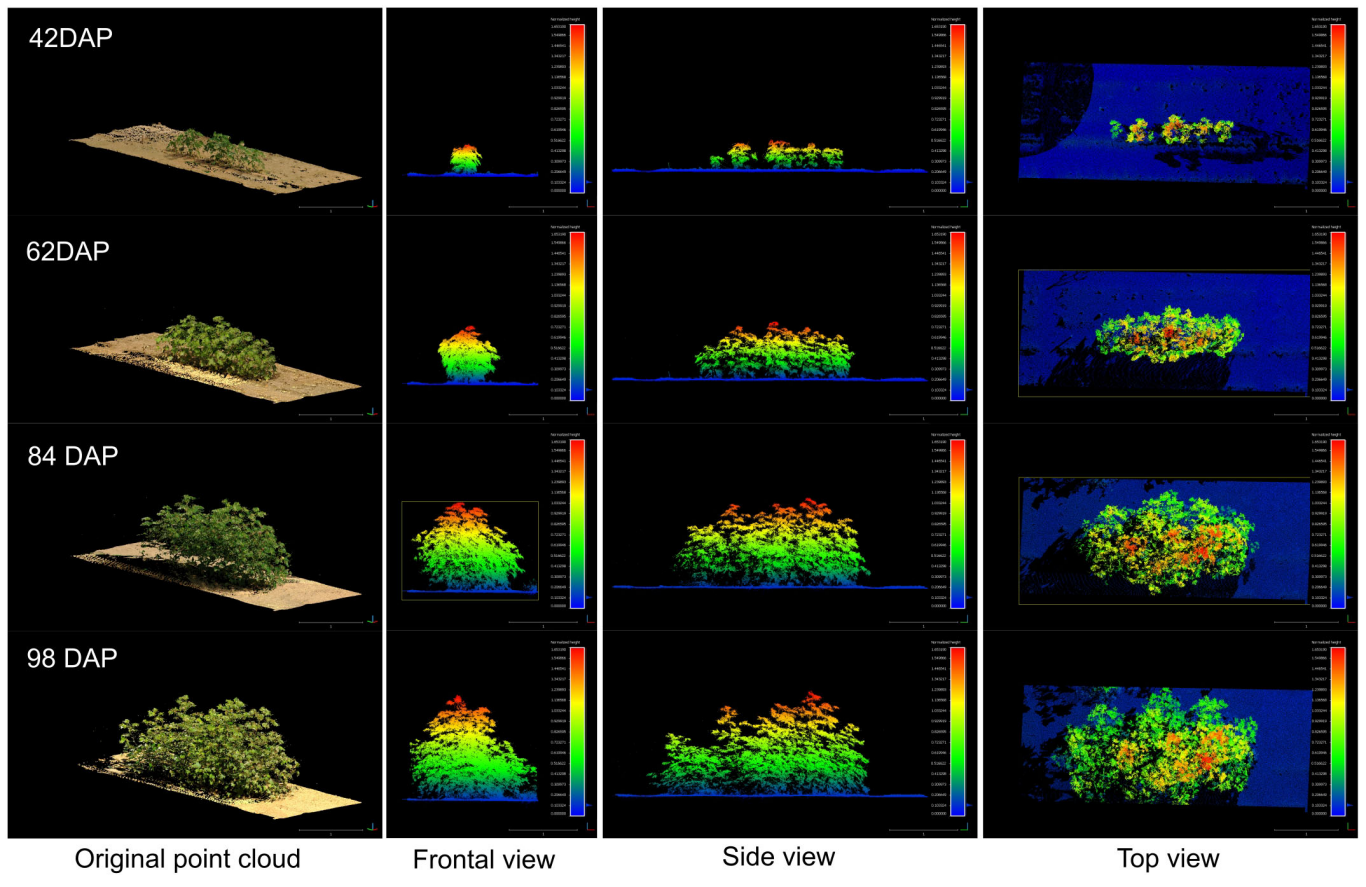
Growth comparison for a sample plot (Plot ID 906) during the vegetative crop growth. The left column shows the original RGB point cloud. The rest of columns show the frontal view, side view, and top view for each respective data collection session colorized by plant height. The figure is color-coded based on plant height, normalized with respect to the terrain (DTM), with blue tones indicating points closer to the ground.

Our methodology for collecting time-series TLS data provided highly detailed 3D information, enabling clear tracking of canopy evolution at the plot level across the distinct crop growth phases (**Figure 12**). The application of multi-scan TLS proved to be a robust technique for crop trait modeling over time, facilitating the reconstruction of detailed 4D models to successfully identify the primary growth phases leading up to crop maturity. The progression of canopy expansion is illustrated in **Supplementary Figure S6**, which shows a detailed profile of the distribution of canopy area per height layer over time for all genotypes. In general, during the initial growth stages, steady stem elongation and leaf area expansion were observed. With increasing resource availability, plant canopy expanded significantly both vertically and horizontally, resulting in exponential leaf area growth for almost all genotypes between 40 and 70 DAP. As the crop approached canopy closure, the canopy height tended to peak, yet vegetative branches continued to develop, significantly increasing lateral growth and the overall occupied volume until reaching canopy closure, where the canopy nears its upper size limit. This approach facilitated the extraction of morphological traits at the plot level and enabled comparative analysis across different genotypes throughout the growing season.

#### 3.6.1 Crop growth model selection

The logistic model consistently emerged as the best-performing model across the analyzed traits, exhibiting the lowest AIC and BIC scores for modeling CH and CV evolution (**Supplementary Table S4**). Notably, the variations in performance among the models were relatively modest, indicating that all selected models provided a reasonably good fit to the data. The ΔAIC and ΔBIC values, which represent the differences in Akaike Information Criterion and Bayesian Information Criterion values, respectively, offer a comparative perspective on each model’s performance relative to the best-fitting model.

In our study, the logistic and 3P-Richards models excelled in estimating CH growth parameters. For CH estimations, the logistic model was closely followed by the 3P-Richards model, with only a 4-point difference. The Gompertz model performed notably worse, with differences exceeding 50 points in both AIC and BIC scores. Conversely, the logistic model outperformed the others in CV growth modeling. Here, the 3P-Richards model had the worst AIC and BIC scores, while the Gompertz model’s performance was intermediate, with AIC and BIC scores 10 points higher than those of the logistic model.

#### 3.6.2 Traits evolution modeling

Our findings revealed significant variability in CH growth parameters across different cotton genotypes, including both maximum CH value and inflection point (**Table 2**). In order to ensure the convergence of the mathematical model and facilitate the estimation of other growth parameters using the available data, we assumed a constant logistic growth rate (*k*) across all genotypes.

**Table 2:** Comparison of logistic growth parameters for canopy height per genotype.

Genotype	k*_CH_*	A*_CH_* ^†^	SE	T*_iCH_* ^†^	SE
Tamcot Sphinx	0.06	1.24*^a^*	0.112	53.3*^abcd^*	1.153
UA 48	0.06	1.26*^a^*	0.097	51.2*^ab^*	0.875
ST5020	0.06	1.40*^ab^*	0.097	49.1*^a^*	0.792
Acala Maxxa	0.06	1.40*^ab^*	0.104	53.2*^abc^*	1.049
DP 1646	0.06	1.48*^ab^*	0.104	55.7*^cde^*	0.918
T0246BC3MDN	0.06	1.58*^abc^*	0.136	53.3*^bcd^*	1.028
DG 3615	0.06	1.59*^ab^*	0.104	52.5*^abc^*	0.941
DP 341	0.06	1.62*^abc^*	0.105	56.1*^cde^*	1.145
DES 56	0.06	1.81*^bc^*	0.104	54.4*^bcd^*	0.804
MDN0101 (GH191)	0.06	1.84*^bc^*	0.099	57.7*^de^*	0.909
T0018MDN	0.06	2.06*^c^*	0.100	59.3*^e^*	0.871

Means and standard error (SE) for maximum height ( $A_{CH}$ ) in meters and inflection point ( $T_{iCH}$ ) in days after planting. The logistic growth rate ( $k_{CH}$ ) was assumed constant for all the genotypes. ^†^Means not sharing any letter are significantly different by the Tukey-test at the 5% level of significance.

The maximum value in a sigmoidal growth curve represents the upper limit or saturation point of growth for the crop. In our analysis, genotype T0018MDN exhibited the highest maximum CH among all genotypes, reaching nearly 2 meters. Genotypes MDN0101 (GH191) and DES 56 closely followed, with a maximum CH of approximately 1.8 meters. In contrast, genotypes Tamcot Sphinx and UA48 were significantly smaller, with maximum CH values around 1.2 meters. The remaining genotypes fell in between, with CH values ranging from 1.4 to 1.6 meters. Notably, ST5020, Acala Maxxa, DP 1646, and DG 3615 had significantly shorter canopies compared to T0018MDN.

The inflection point is a critical feature that marks the change in crop growth dynamics. This point, where the curve’s slope is at its maximum, represents the stage where the rate of growth transitions from being exponential to linear, signifying the phase of most rapid change in the growth rate. In our experiment, T0018MDN genotype was the last in reaching maximum growth rate, occurring after 59 DAP (T*_i_* = 59.3 DAP). This was significantly later than the other genotypes, except for MDN0101 (GH191), DP 341, and DP 1646. In contrast, ST5020 and UA48 took around 50 days to reach their maximum growth rates. The rest of the genotypes exhibited inflection points of 53 DAP or more. Notably, MDN0101 (GH191) was significantly more slowly in reaching its peak than DG 3625, Acala Maxxa, UA48, and ST5020, taking approximately 54 DAP.

Our analysis of CV estimations over time revealed substantial genotype-dependent differences in CV growth (**Table 3**). Similar to the CH analysis, we assumed a constant logistic growth rate (*k*) for all genotypes to ensure model convergence. Among the genotypes assessed, T0018MDN exhibited the highest plant volume, surpassing 7 m^3^. Following closely were genotypes DG 3615, DES 56, and DP 341, each with volumes exceeding 6 m^3^. In contrast, Tamcot Sphinx attained a maximum volume of less than 3 m^3^, significantly smaller than T0018MDN. The remaining genotypes displayed CV values ranging between 3 and 7 m^3^. Regarding the inflection point for CV growth modeling, genotype DP 1646 demonstrated the highest value among all genotypes, occurring at approximately 70 DAP. Most other genotypes exhibited inflection points between 64 and 68 DAP. MDN0101 (GH191) emerged as the most precocious in reaching its maximum growth rate, at around 63 DAP, indicating a significant earlier maturation compared to genotypes T0018MDN, DG 3615, and DP 341.

**Table 3:** Comparison of logistic growth parameters for canopy volume (CV) per genotype.

Genotype	k*_CV_*	A*_CV_* ^†^	SE	T*_iCV_* ^†^	SE
Tamcot Sphinx	0.106	2.76*^a^*	0.92	64.4*^abc^*	1.435
UA 48	0.106	3.68*^ab^*	0.80	64.8*^ab^*	1.058
Acala Maxxa	0.106	4.28*^ab^*	0.86	64.0*^ab^*	1.116
ST5020	0.106	4.28*^ab^*	0.80	65.3*^abc^*	1.139
DP 1646	0.106	4.77*^ab^*	0.85	70.1*^c^*	1.157
T0246BC3MDN	0.106	4.84*^ab^*	1.12	66.1*^abc^*	1.308
MDN0101 (GH191)	0.106	5.40*^ab^*	0.80	62.6*^a^*	0.846
DP 341	0.106	6.45*^ab^*	0.85	68.3*^bc^*	1.218
DES 56	0.106	6.49*^ab^*	0.85	66.7*^abc^*	0.962
DG 3615	0.106	6.67*^ab^*	0.85	67.4*^bc^*	1.087
T0018MDN	0.106	7.25*^b^*	0.79	67.0*^bc^*	0.849

Means and standard error (SE) for maximum canopy volume (A_CV)_ in cubic meters and inflection point (T_iCV)_ in days after planting. The logistic growth rate (k_CV)_ was assumed constant for all the genotypes. ^†^Means not sharing any letter are significantly different by the Tukey-test at the 5% level of significance.

**Figure 13:**
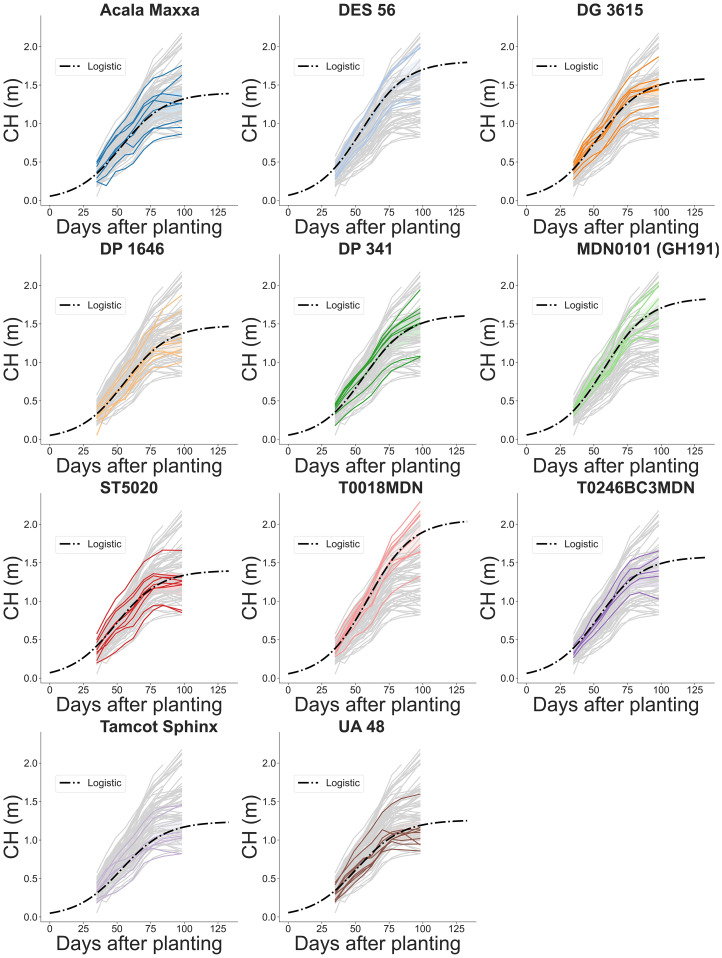
Temporal evolution of plot-level canopy height (CH) across the growing season for 11 genotypes. Grey lines indicate estimated CH values for all plots in the field. Colored solid lines highlight the estimated CH for a specific genotype. Dash-dotted lines depict fitted logistic growth curves.

**Figure 14:**
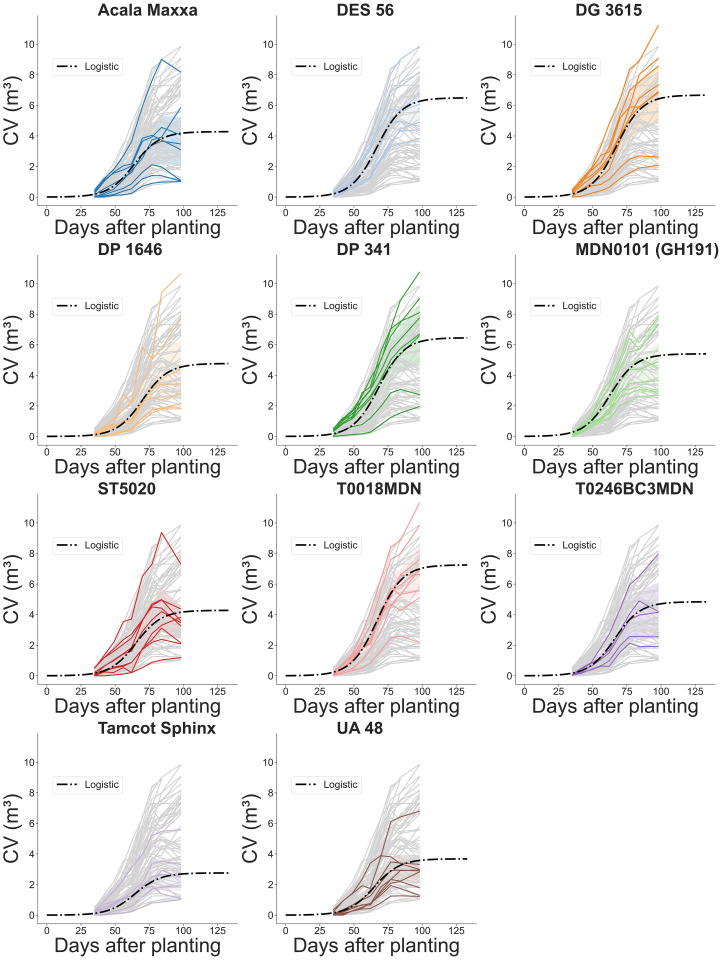
Temporal evolution of plot-level canopy volume (CV) during the growing season for 11 genotypes. Grey lines represent estimated CV values for every plot computed using the method based on projected area. Colored solid lines highlight the estimated CV of individual plots for a specific genotype. Dash-dotted lines depict fitted logistic growth curves.

The complex interplay between genotype, environment, and crop development is evident in the diverse morphological traits analyzed and the corresponding evolution curves of CH (**Figure 13**) and CV (**Figure 14**). The evolution of both CH and CV reflects the gradual pace of growth of the crop during the initial stages. As the season progressed and resources become more available, plant canopy expanded both in height and laterally, resulting in exponential CV growth across all genotypes between 40 and 70 DAP. This period of rapid development indicated the crop’s increasing capacity to capture light energy. Around 75 DAP, a visible slowdown in stem elongation is observed across nearly all genotypes, signaling the approach to canopy closure. This phenomenon suggests that the crop is nearing its upper size limit, with further CH growth becoming increasingly constrained. Tracking growth traits over time provides valuable insights into the dynamic evolution of cotton crop development, including genotype-specific responses to environmental cues and management practices. These insights can guide breeders and researchers in selecting genotypes with desired traits to improve yield, enhance pest resistance, and ensure adaptability to diverse growing conditions.

## 4 Discussion

### 4.1 Time-series TLS data for field phenotyping

Using time-related data in plant phenotyping represents a valuable approach to gaining a deeper understanding of the temporal dynamics of plant growth. Plants respond to changing environmental conditions, and field data collected over time can capture these variations, providing continuous insights into trait evolution during the course of a growing season. This can allow for a better understanding of crop development (Pauli et al., 2016), holding promise for advancing our knowledge of plant biology and supporting the development of crops adapted to varying environmental conditions (Miao et al., 2020). In plant breeding, this information can be key to support genotype selection based on new traits that allow for a more efficient use of resources (Li et al., 2022).

Traditional plant phenotyping methods often limit analyses to a single time point, potentially overlooking vital changes and interactions between the crop and its environment during critical developmental stages (Tardieu et al., 2017). Unlike conventional static measurements, time-series data are composed of a sequence of data collected at different timestamps. This temporal context facilitates the identification of key stages of development and growth patterns, offering valuable information about the rate of growth, periods of stability, and potential stress responses (Chen et al., 2014).

Integrating time-series data with advanced 3D technologies like LiDAR enhances phenotyping for precision agriculture. LiDAR technology has contributed to the advancement of field phenotyping, offering direct access to complex 3D morphological trait information and allowing for detailed reconstructions of plant structures (Guo et al., 2018). Time-series TLS data can improve the accuracy of growth models by capturing intricate details of plant development during the whole growing season (Jin et al., 2021b). Our work demonstrated that TLS time-series data can provide consistent information on traits such as canopy height and volume, enabling precise crop traits tracking. This information can increase our understanding of critical growth parameters to optimize management strategies and make more informed decisions in crop breeding.

### 4.2 Importance of 4D registration of TLS data for crop growth tracking

Detailed 4D models, incorporating the temporal dimension into 3D spatial data from TLS scans, provide a powerful tool for analyzing and tracking morphological traits across the growing season. However, generating these models poses unique challenges, primarily from the need to align repeated measurements over time. The complex transformations that crops undergo during the growing season complicate the registration of point clouds collected at different time points. Ensuring the consistency and reliability of TLS data collection and processing becomes essential in field conditions (Pieruschka and Schurr, 2019).

Accurate data alignment is crucial for tracking changes in plant structure. While some methods use fixed targets to aid co-registration (Tilly et al., 2014; Friedli et al., 2016; Su et al., 2019), this approach can be labor-intensive and error-prone. The consistent positioning of targets in agricultural fields, where machinery needs to operate or other experiments need to be executed, can be problematic. Deviations in the target placement between surveys can impact the temporal alignment accuracy, and hence the estimation of traits over time.

Inspired by the positive results from a previous study in maize, soybean, and wheat using semi-permanent targets (Friedli et al., 2016), we initially adopted this concept to benchmark our methodology. This study reported deviations in the positions of spherical targets between 2.5 mm and 10 mm. However, after preliminary processing of data from our initial collection sessions, we found deviations in target locations exceeding 20 mm. These larger deviations were not due to the registration process itself but were likely caused by external factors beyond our control. Our experimental field was also used for other studies involving autonomous robot navigation, and areas with heavy foot and tractor traffic seemed to impact the soil around the spheres, causing the structures to shift.

To address this issue, we proposed an innovative approach that relies solely on the collected data for registration, reducing dependence on artificial targets and minimizing the impact of such external factors. By not relying on physical markers that can be displaced, our approach maintains accuracy over long periods and in environments subject to change. This flexibility makes it especially suitable for dynamic field conditions where traditional fixed targets might fail to provide consistent accuracy. This streamlines field setups and ensures consistent and reproducible results.

Our approach has demonstrated effectiveness in overcoming these challenges, offering an accessible and robust methodology for TLS-based phenotyping in dynamic field environments. Unlike approaches requiring continuous acquisition for ensuring common features for registration (Li et al., 2023), our multi-step TLS data alignment leveraged invariant elements naturally present in the collected point clouds to roughly align them. This allowed us to decouple the registration process from changing elements such as the crop, facilitating the registration of point clouds collected at distant time intervals. The observed registration errors, comparable to expected errors in rigid registration at the organ level (Chebrolu et al., 2021) and falling within the range of RMSE values for height estimation, indicate the accuracy of our approach in capturing time-series TLS data. This highlights the potential of our method for LiDAR-based crop phenotyping under dynamic field conditions.

### 4.3 Challenges in growth modeling for crop breeding

Understanding the growth patterns of cotton plants is essential for effective crop management and informed breeding strategies (Ritchie et al., 2007). Cotton plant development typically follows a sigmoid function, characterized by slow initial growth during establishment, followed by exponential vegetative growth that gradually slows as the crop approaches canopy closure (Snider et al., 2021). Growth modeling plays a key role in estimating key parameters defining these growth curves (Gregorczyk, 1998), providing a systematic approach to incorporate insights into crop phenotyping (Costa et al., 2019).

Growth modeling from field data can be challenging, and many of the growth parameters extracted with our methodology could not be obtained any other way on a large scale. Focused on the analysis of three key morphological traits, CH, CA, and CV, across diverse cotton genotypes, our study unraveled the dynamics of cotton growth and the relationships between growth parameters, providing valuable insights for genotype selection tailored to specific requirements. However, fitting maximal models with all random effects may fail to converge because the random effects structure has a complexity not supported by the underlying data (Barr et al., 2013). Simplification of the random effects structure can help the model to converge (Bates et al., 2015).

Trade-offs are often necessary due to computational limitations and the need for efficient model fitting (McCrea et al., 2023). Challenges may arise across the data analysis pipeline, including processing, model development, and information extraction. Fitting maximal models that include all random effects in growth parameters can face convergence challenges due to the complexity of the random effects structure (Barr et al., 2013). Our study revealed that growth rate could be one of the most complex parts of the random effects structure for crop growth modeling. Primarily, we dropped covariance terms for some of the random effects, as suggested in one paper (Seedorff et al., 2019) to try to achieve convergence. However, it was not sufficient for our model to converge, and we opted for a more drastic approach of fixing the growth rate for all the genotypes, removing the slope entirely, in order to reduce the complexity of our model. This led us to simplify the model by fixing the growth rate for all genotypes, emphasizing the importance of balancing model complexity and convergence (Bates et al., 2015).

### 4.4 Limitations and future work

The adaptability of TLS technology to capture 3D structural information makes it inherently suitable for studying a wide range of plant species. While our methodology has demonstrated effectiveness in a cotton breeding field, highlighting its potential application in similar agricultural contexts, its generalizability across diverse crops and environmental conditions remains to be fully explored.

Additionally, uncertainties in the use of estimated CV as a proxy for estimating canopy light interception should be acknowledged. Variability in environmental conditions can affect the accuracy of IPAR*_f_* measurements and validating its relationship with our CV estimation method warrants further research. Understanding these uncertainties is key for enhancing the reliability and applicability of our methodology across different agricultural settings.

Further experimentation across different field settings is imperative to comprehensively assess the adaptability and generalizability of our methodology. Expanding these efforts to accommodate various research environments will be key to establishing a scalable methodology for consistent field phenotyping over time. Understanding how factors such as canopy structure, plant density, and environmental influences interact with our methodology is crucial for evaluating its scalability and robustness across different crop types and field conditions. Our initial findings indicated that our methodology could create accurate 4D crop models under conditions of minimal plant overlap. However, maintaining model accuracy became challenging as canopy density increased. Different crop types may require tailored strategies for BEV-based alignment due to their unique growth characteristics. For example, maize plants, which are individually planted and in early stages may project only a small footprint, may require a more precise clustering process. Similarly, densely planted crops like wheat can introduce additional complexities in data interpretation and analysis.

Despite the effectiveness and accuracy achieved in our cotton field, challenges such as laser beam shadowing and occlusions were not entirely eliminated, particularly in advanced growth stages. These issues are common in many remote and proximal sensing studies conducted in the field, demanding further research for resolution. The complex structure of crops like cotton poses a challenge, where increasing the number of scan locations for multi-scan TLS-based field analysis may not completely eliminate occlusion effects. Striking a balance between the need for more scans and efficient resource utilization, including time and computing power, necessitates thorough site studies for TLS site planning to optimize data collection. We are actively exploring the potential of physics-based simulators for knowledge-guided TLS site planning. These simulations can provide valuable insights into the optimal distribution of scan locations, especially in complex field environments, enhancing the efficiency of TLS-based field phenotyping.

Moreover, the increased number of scan locations introduces new challenges, particularly in terms of time consumption and logistics. Traditional practices for LiDAR-based scanning involve manually moving the scanner from one scan location to another, contributing to the time-intensive nature of data collection. In a prior study, we demonstrated the potential use of a ground robot to automate TLS data acquisition in a breeding field (Rodriguez-Sanchez and Li, 2022). We are working on improving this system to autonomously determine the number of scans and their distribution throughout the field, thereby fully automating the TLS-based phenotyping process. This work is currently under review in a reputed journal, and a preprint is available on arXiv (Rodriguez-Sanchez et al., 2024). This innovative approach aims to enhance the efficiency of TLS data collection for plant breeding, streamlining the phenotyping workflow and aligning with broader trends in automation and robotics within the field of agriculture.

## 5 Conclusions

In this study, we introduced an innovative methodology for precisely registering point clouds collected under field conditions, enabling LiDAR-based crop phenotyping over time. This work emphasizes the critical importance of precise point cloud data collection, accurate registration, and precise modeling for TLS-based field phenotyping. Our two-phase TLS data registration approach has demonstrated its effectiveness in aligning point clouds captured up to two months apart during the vegetative growth season, significantly reducing alignment errors. By leveraging terrain points and crop row distribution, our method provides a reliable and efficient solution for monitoring crop morphological growth, enabling breeders to consistently acquire accurate phenotypic traits from the same physical locations at different time points.

As digital technologies advance, the refinement of current procedures for in-field data collection and processing will strengthen our ability to enhance crop improvement in a more efficient manner. Despite challenges such as late-season occlusions, our approach presents a promising solution to enhance cotton breeding programs by offering a reliable digital approach to monitor traits over time. It provides a foundation for informed decision-making and genotype selection based on desirable growth characteristics. The findings of this study can contribute significantly to understanding cotton plant growth and genotype variations, offering valuable insights for optimizing cotton crop management and advancing plant phenomics.

## Data availability statement

The raw data supporting the conclusions of this article will be made available by the authors, without undue reservation.

## Author contributions

JR-S: Conceptualization, Data curation, Formal analysis, Investigation, Methodology, Software, Validation, Visualization, Writing – original draft, Writing – review & editing. JS: Methodology, Resources, Writing – review & editing. KJ: Funding acquisition, Methodology, Project administration, Resources, Supervision, Writing – review & editing. CL: Conceptualization, Funding acquisition, Methodology, Project administration, Resources, Supervision, Writing – original draft, Writing – review & editing.

## References

[B1] Akaike H. (1992). Information theory and an extension of the maximum likelihood principle. In Springer series in statistics (New York, NY: Springer New York), 610–624. doi: 10.1007/978-1-4612-0919-538

[B2] Baker D. N. Meyer R. E. (1966). Influence of stand geometry on light interception and net photosynthesis in cotton. Crop Sci. 6, 15–19. doi: 10.2135/cropsci1966.0011183X000600010004x

[B3] Barr D. J. Levy R. Scheepers C. Tily H. J. (2013). Random effects structure for confirmatory hypothesis testing: Keep it maximal. J. Memory Lang. 68, 255–278. doi: 10.1016/j.jml.2012.11.001 PMC3881361 24403724

[B4] Bates D. M. Kliegl R. Vasishth S. Baayen H. (2015). Parsimonious mixed models. ArXiv. doi: 10.48550/arXiv.1506.04967

[B5] Besl P. McKay N. D. (1992). A method for registration of 3-D shapes. IEEE Trans. Pattern Anal. Mach. Intell. 14, 239–256. doi: 10.1109/34.121791

[B6] Carlone L. Dong J. Fenu S. Rains G. Dellaert F. (2015). Towards 4D crop analysis in precision agriculture: Estimating plant height and crown radius over time via expectation-maximization. In ICRA workshop on robotics in agriculture. (Seattle, WA, USA: International Conference on Robotics and Automation (ICRA)).

[B7] Chawade A. van Ham J. Blomquist H. Bagge O. Alexandersson E. Ortiz R. (2019). Highthroughput field-phenotyping tools for plant breeding and precision agriculture. Agronomy 9, 258. doi: 10.3390/agronomy9050258

[B8] Chebrolu N. Labe T. Stachniss C. (2018). Robust long-term registration of UAV images of crop fields for precision agriculture. IEEE Robotics Automation Lett. 3, 3097–3104. doi: 10.1109/LRA.2018.2849603

[B9] Chebrolu N. Labe T. Stachniss C. (2020). Spatio-temporal non-rigid registration of 3D point clouds of plants. In 2020 IEEE International Conference on Robotics and Automation (ICRA). (Paris, France: IEEE), 3112–3118. doi: 10.1109/icra40945.2020.9197569

[B10] Chebrolu N. Magistri F. Läbe T. Stachniss C. (2021). Registration of spatio-temporal point clouds of plants for phenotyping. PloS One 16, e0247243. doi: 10.1371/journal.pone.0247243 33630896 PMC7906482

[B11] Chen D. Neumann K. Friedel S. Kilian B. Chen M. Altmann T.. (2014). Dissecting the phenotypic components of crop plant growth and drought responses based on high-throughput image analysis. Plant Cell 26, 4636–4655. doi: 10.1105/tpc.114.129601 25501589 PMC4311194

[B12] Costa J. M. Marques da Silva J. Pinheiro C. Barón M. Mylona P. Centritto M.. (2019). Opportunities and limitations of crop phenotyping in Southern European countries. Front. Plant Sci. 10. doi: 10.3389/fpls.2019.01125 PMC6774291 31608085

[B13] Crommelinck S. Höfle B. (2016). Simulating an autonomously operating low-cost static terrestrial LiDAR for multitemporal maize crop height measurements. Remote Sens. 8, 205. doi: 10.3390/rs8030205

[B14] Dong J. Burnham J. G. Boots B. Rains G. Dellaert F. (2017). 4D crop monitoring: Spatio-temporal reconstruction for agriculture. In 2017 IEEE International Conference on Robotics and Automation (ICRA). (Singapore: IEEE), 3878–3885. doi: 10.1109/icra.2017.7989447

[B15] Eitel J. U. Magney T. S. Vierling L. A. Greaves H. E. Zheng G. (2016). An automated method to quantify crop height and calibrate satellite-derived biomass using hypertemporal LiDAR. Remote Sens. Environ. 187, 414–422. doi: 10.1016/j.rse.2016.10.044

[B16] Ermanis A. Gobbo S. Snider J. L. Cohen Y. Liakos V. Lacerda L.. (2020). Defining physiological contributions to yield loss in response to irrigation in cotton. J. Agron. Crop Sci. 207, 186–196. doi: 10.1111/jac.12453

[B17] Fix E. Hodges J. L. (1951). Discriminatory analysis: Nonparametric discrimination: Consistency properties. (Randolph Field, TX: USAF School of Aviation Medicine). doi: 10.1037/e471672008-001

[B18] Friedli M. Kirchgessner N. Grieder C. Liebisch F. Mannale M. Walter A. (2016). Terrestrial 3D laser scanning to track the increase in canopy height of both monocot and dicot crop species under field conditions. Plant Methods 12, 1–15. doi: 10.1186/s13007-016-0109-7 26834822 PMC4731982

[B19] Furbank R. T. Tester M. (2011). Phenomics – technologies to relieve the phenotyping bottleneck. Trends Plant Sci. 16, 635–644. doi: 10.1016/j.tplants.2011.09.005 22074787

[B20] Gelard W. Herbulot A. Devy M. Casadebaig P. (2018). 3D leaf tracking for plant growth monitoring. In 2018 25th IEEE International Conference on Image Processing (ICIP). (Athens, Greece: IEEE), 3663–3667. doi: 10.1109/icip.2018.8451553

[B21] Gregorczyk A. (1998). Richards plant growth model. J. Agron. Crop Sci. 181, 243–247. doi: 10.1111/j.1439-037X.1998.tb00424.x

[B22] Großkinsky D. K. Svensgaard J. Christensen S. Roitsch T. (2015). Plant phenomics and the need for physiological phenotyping across scales to narrow the genotype-to-phenotype knowledge gap. J. Exp. Bot. 66, 5429–5440. doi: 10.1093/jxb/erv345 26163702

[B23] Günder M. Ispizua Yamati F. R. Kierdorf J. Roscher R. Mahlein A.-K. Bauckhage C. (2022). Agricultural plant cataloging and establishment of a data framework from UAV-based crop images by computer vision. GigaScience 11. doi: 10.1093/gigascience/giac054 PMC9205758 35715875

[B24] Guo T. Fang Y. Cheng T. Tian Y. Zhu Y. Chen Q.. (2019). Detection of wheat height using optimized multi-scan mode of LiDAR during the entire growth stages. Comput. Electron. Agric. 165, 104959. doi: 10.1016/j.compag.2019.104959

[B25] Guo Q. Wu F. Pang S. Zhao X. Chen L. Liu J.. (2018). Crop 3D-a LiDAR based platform for 3D high-throughput crop phenotyping. Sci. China. Life Sci. 61, 328—339. doi: 10.1007/s11427-017-9056-0 28616808

[B26] Herrero-Huerta M. Lindenbergh R. Gard W. (2018). Leaf movements of indoor plants monitored by terrestrial LiDAR. Front. Plant Sci. 9. doi: 10.3389/fpls.2018.00189 PMC5829619 29527217

[B27] Hosoi F. Omasa K. (2009). Estimating vertical plant area density profile and growth parameters of a wheat canopy at different growth stages using three-dimensional portable LiDAR imaging. ISPRS J. Photogrammetry Remote Sens. 64, 151–158. doi: 10.1016/j.isprsjprs.2008.09.003

[B28] Hosoi F. Omasa K. (2012). Estimation of vertical plant area density profiles in a rice canopy at different growth stages by high-resolution portable scanning LiDAR with a lightweight mirror. ISPRS J. Photogrammetry Remote Sens. 74, 11–19. doi: 10.1016/j.isprsjprs.2012.08.001

[B29] Hui F. Zhu J. Hu P. Meng L. Zhu B. Guo Y.. (2018). Image-based dynamic quantification and high-accuracy 3D evaluation of canopy structure of plant populations. Ann. Bot. 121, 1079–1088. doi: 10.1093/aob/mcy016 29509841 PMC5906925

[B30] Jiménez-Berni J. A. Deery D. M. Rozas-Larraondo P. Condon A. G. Rebetzke G. J. James R.. (2018). High throughput determination of plant height, ground cover, and above-ground biomass in wheat with LiDAR. Front. Plant Sci. 9. doi: 10.3389/fpls.2018.00237 PMC5835033 29535749

[B31] Jin S. Su Y. Zhang Y. Song S. Li Q. Liu Z.. (2021a). Exploring seasonal and circadian rhythms in structural traits of field maize from LiDAR time series. Plant Phenomics 2021. doi: 10.34133/2021/9895241 PMC8441379 34557676

[B32] Jin S. Sun X. Wu F. Su Y. Li Y. Song S.. (2021b). LiDAR sheds new light on plant phenomics for plant breeding and management: Recent advances and future prospects. ISPRS J. Photogrammetry Remote Sens. 171, 202–223. doi: 10.1016/j.isprsjprs.2020.11.006

[B33] Kaur N. Snider J. L. Paterson A. H. Grey T. L. Li C. Virk G.. (2023). Variation in thermotolerance of photosystem II energy trapping, intersystem electron transport, and photosystem i electron acceptor reduction for diverse cotton genotypes. Plant Physiol. Biochem. 201, 107868. doi: 10.1016/j.plaphy.2023.107868 37459803

[B34] Kim J. Y. (2020). Roadmap to high throughput phenotyping for plant breeding. J. Biosyst. Eng. 45, 43–55. doi: 10.1007/s42853-020-00043-0

[B35] Lenth R. V. (2023). Emmeans: Estimated marginal means, aka least-squares means, R package version 1.8.8. Retrieved from https://CRAN.R-project.org/package=emmeans

[B36] Li D. Bai D. Tian Y. Li Y.-H. Zhao C. Wang Q.. (2022). Time series canopy phenotyping enables the identification of genetic variants controlling dynamic phenotypes in soybean. J. Integr. Plant Biol. 65, 117–132. doi: 10.1111/jipb.13380 36218273

[B37] Li Y. Fan X. Mitra N. J. Chamovitz D. Cohen-Or D. Chen B. (2013). Analyzing growing plants from 4D point cloud data. ACM Trans. Graphics 32, 1–10. doi: 10.1145/2508363.2508368

[B38] Li Y. Wen W. Fan J. Gou W. Gu S. Lu X.. (2023). Multi-source data fusion improves time-series phenotype accuracy in maize under a field high-throughput phenotyping platform. Plant Phenomics 5, 0043. doi: 10.34133/plantphenomics.0043 37223316 PMC10202381

[B39] Lin Y. (2015). LiDAR: An important tool for next-generation phenotyping technology of high potential for plant phenomics? Comput. Electron. Agric. 119, 61–73. doi: 10.1016/j.compag.2015.10.011

[B40] Loomis R. Williams W. (1969). Productivity and the morphology of crop stands: Patterns with leaves. In Physiological aspects of crop yield. Eds. Eastin J. D. Haskins F. A. Sullivan C. Y. van Bavel C. H. M. (Madison, WI: American Society of Agronomy, Crop Science Society of America), 27–47. doi: 10.2135/1969.physiologicalaspects.c3

[B41] Madec S. Baret F. de Solan B. Thomas S. Dutartre D. Jézéquel S.. (2017). High-throughput phenotyping of plant height: Comparing unmanned aerial vehicles and ground LiDAR estimates. Front. Plant Sci. 8. doi: 10.3389/fpls.2017.02002 PMC5711830 29230229

[B42] Magistri F. Chebrolu N. Stachniss C. (2020). Segmentation-based 4D registration of plants point clouds for phenotyping. In 2020 IEEE/RSJ International Conference on Intelligent Robots and Systems (IROS). (Las Vegas, NV, USA: IEEE), 2433–2439. doi: 10.1109/iros45743.2020.9340918

[B43] Malambo L. Popescu S. Horne D. Pugh N. Rooney W. (2019). Automated detection and measurement of individual sorghum panicles using density-based clustering of terrestrial LiDAR data. ISPRS J. Photogrammetry Remote Sens. 149, 1–13. doi: 10.1016/j.isprsjprs.2018.12.015

[B44] Malambo L. Popescu S. Murray S. Putman E. Pugh N. Horne D.. (2018). Multitemporal field-based plant height estimation using 3D point clouds generated from small unmanned aerial systems high-resolution imagery. Int. J. Appl. Earth Observation Geoinformation 64, 31–42. doi: 10.1016/j.jag.2017.08.014

[B45] McCrea R. King R. Graham L. Börger L. (2023). Realising the promise of large data and complex models. Methods Ecol. Evol. 14, 4–11. doi: 10.1111/2041-210X.14050

[B46] Medic T. Bömer J. Paulus S. (2023). Challenges and recommendations for 3D plant phenotyping in agriculture using terrestrial lasers scanners. ISPRS Ann. Photogrammetry Remote Sens. Spatial Inf. Sci. X-1/W1-2023, 1007–1014. doi: 10.5194/isprs-annals-X-1-W1-2023-1007-2023. X-1/W1-2023.

[B47] Miao C. Xu Y. Liu S. Schnable P. S. Schnable J. C. (2020). Increased power and accuracy of causal locus identification in time series genome-wide association in sorghum. Plant Physiol. 183, 1898–1909. doi: 10.1104/pp.20.00277 32461303 PMC7401099

[B48] Paproki A. Sirault X. Berry S. Furbank R. Fripp J. (2012). A novel mesh processing based technique for 3D plant analysis. BMC Plant Biol. 12, 63. doi: 10.1186/1471-2229-12-63 22553969 PMC3464618

[B49] Pauli D. Andrade-Sanchez P. Carmo-Silva A. E. Gazave E. French A. N. Heun J.. (2016). Field-based high-throughput plant phenotyping reveals the temporal patterns of quantitative trait loci associated with stress-responsive traits in cotton. G3 Genes—Genomes—Genetics 6, 865–879. doi: 10.1534/g3.115.023515 26818078 PMC4825657

[B50] Paulus S. Schumann H. Kuhlmann H. Léon J. (2014). High-precision laser scanning system for capturing 3D plant architecture and analysing growth of cereal plants. Biosyst. Eng. 121, 1–11. doi: 10.1016/j.biosystemseng.2014.01.010

[B51] Pieruschka R. Schurr U. (2019). Plant phenotyping: Past, present, and future. Plant Phenomics 2019. doi: 10.34133/2019/7507131 PMC7718630 33313536

[B52] Pinheiro J. C. Bates D. M. (2000). Mixed-effects models in S and S-PLUS (New York: Springer). doi: 10.1007/b98882

[B53] Pokhrel A. Snider J. L. Virk S. Sintim H. Y. Hand L. C. Vellidis G.. (2023). Quantifying physiological contributions to nitrogen-induced yield variation in field-grown cotton. Field Crops Res. 299, 108976. doi: 10.1016/j.fcr.2023.108976

[B54] R Core Team (2023). R: A language and environment for statistical computing (Vienna, Austria: R Foundation for Statistical Computing).

[B55] Ritchie G. L. Bednarz C. W. Jost P. H. Brown S. M. (2007). Cotton growth and development. In Bulletin 1952 (Athens, GA: University of Georgia).

[B56] Rodriguez-Sanchez J. Johnsen K. Li C. (2024). A ground mobile robot for autonomous terrestrial laser scanning-based field phenotyping. ArXiv. doi: 10.48550/arXiv.2404.04404

[B57] Rodriguez-Sanchez J. Li C. (2022). An autonomous ground system for 3D LiDAR-based crop scouting. In 2022 Houston, TX July 17-20, 2022 (St. Joseph, MI: American Society of Agricultural and Biological Engineers), 1–10. doi: 10.13031/aim.202200142

[B58] Rohlf F. J. Slice D. (1990). Extensions of the procrustes method for the optimal superimposition of landmarks. Systematic Zoology 39, 40. doi: 10.2307/2992207

[B59] Rote G. (1991). Computing the minimum Hausdorff distance between two point sets on a line under translation. Inf. Process. Lett. 38, 123–127. doi: 10.1016/0020-0190(91)90233-8

[B60] Schwarz G. (1978). Estimating the dimension of a model. Ann. Stat 6, 461–464. doi: 10.1214/aos/1176344136

[B61] Seedorff M. Oleson J. McMurray B. (2019). Maybe maximal: Good enough mixed models optimize power while controlling type I error. PsyArXiv. doi: 10.31234/osf.io/xmhfr

[B62] Singh A. Ganapathysubramanian B. Singh A. K. Sarkar S. (2016). Machine learning for high-throughput stress phenotyping in plants. Trends Plant Sci. 21, 110–124. doi: 10.1016/j.tplants.2015.10.015 26651918

[B63] Sneath P. H. A. (1967). Trend-surface analysis of transformation grids. J. Zoology 151, 65–122. doi: 10.1111/j.1469-7998.1967.tb02866.x

[B64] Snider J. Bange M. Heitholt J. (2021). Cotton. In Crop physiology case histories for major crops (Cambridge, MA: Elsevier), 714–746. doi: 10.1016/b978-0-12-819194-1.00022-0

[B65] Su Y. Wu F. Ao Z. Jin S. Qin F. Liu B.. (2019). Evaluating maize phenotype dynamics under drought stress using terrestrial LiDAR. Plant Methods 15 (11). doi: 10.1186/s13007-019-0396-x PMC6360786 30740137

[B66] Sun S. Li C. Chee P. W. Paterson A. H. Meng C. Zhang J.. (2021). High resolution 3D terrestrial LiDAR for cotton plant main stalk and node detection. Comput. Electron. Agric. 187, 106276. doi: 10.1016/j.compag.2021.106276

[B67] Sun S. Li C. Paterson A. (2017). In-field high-throughput phenotyping of cotton plant height using LiDAR. Remote Sens. 9, 377. doi: 10.3390/rs9040377 PMC5786533 29403522

[B68] Sun S. Li C. Paterson A. H. Jiang Y. Xu R. Robertson J. S.. (2018). In-field high throughput phenotyping and cotton plant growth analysis using LiDAR. Front. Plant Sci. 9. doi: 10.3389/fpls.2018.00016 PMC5786533 29403522

[B69] Tao H. Xu S. Tian Y. Li Z. Ge Y. Zhang J.. (2022). Proximal and remote sensing in plant phenomics: 20 years of progress, challenges, and perspectives. Plant Commun. 3, 100344. doi: 10.1016/j.xplc.2022.100344 35655429 PMC9700174

[B70] Tardieu F. Cabrera-Bosquet L. Pridmore T. Bennett M. (2017). Plant phenomics, from sensors to knowledge. Curr. Biol. 27, R770–R783. doi: 10.1016/j.cub.2017.05.055 28787611

[B71] Tilly N. Hoffmeister D. Cao Q. Huang S. Lenz-Wiedemann V. Miao Y.. (2014). Multitemporal crop surface models: accurate plant height measurement and biomass estimation with terrestrial laser scanning in paddy rice. J. Appl. Remote Sens. 8, 83671. doi: 10.1117/1.JRS.8.083671

[B72] Tjørve E. Tjørve K. M. (2010). A unified approach to the Richards-model family for use in growth analyses: Why we need only two model forms. J. Theor. Biol. 267, 417–425. doi: 10.1016/j.jtbi.2010.09.008 20831877

[B73] Voss-Fels K. P. Stahl A. Hickey L. T. (2019). Q&A: Modern crop breeding for future food security. BMC Biol. 17, 1–7. doi: 10.1186/s12915-019-0638-4 30803435 PMC6390336

[B74] Wang X. Singh D. S. K. Marla S. R. Morris G. P. Poland J. A. (2018). Field-based high-throughput phenotyping of plant height in sorghum using different sensing technologies. Plant Methods 14 (53). doi: 10.1186/s13007-018-0324-5 PMC6031187 29997682

[B75] Xavier A. Hall B. Hearst A. A. Cherkauer K. A. Rainey K. M. (2017). Genetic architecture of phenomic-enabled canopy coverage in glycine max. Genetics 206, 1081–1089. doi: 10.1534/genetics.116.198713 28363978 PMC5499164

[B76] Zhang X. Pourreza A. Cheung K. H. Zuniga-Ramirez G. Lampinen B. D. Shackel K. A. (2021). Estimation of fractional photosynthetically active radiation from a canopy 3D model; case study: Almond yield prediction. Front. Plant Sci. 12. doi: 10.3389/fpls.2021.715361 PMC8427806 34512697

[B77] Zhou X. Wang P. Tansey K. Zhang S. Li H. Tian H. (2020). Reconstruction of time series leaf area index for improving wheat yield estimates at field scales by fusion of sentinel-2, -3 and MODIS imagery. Comput. Electron. Agric. 177, 105692. doi: 10.1016/j.compag.2020.105692

