# Supplementary material for: Cotton morphological traits tracking through spatiotemporal registration of terrestrial laser scanning time-series data
Source: Front Plant Sci. 2024 Aug 1;15:1436120. doi: 10.3389/fpls.2024.1436120 (PMC11325728; doi:10.3389/fpls.2024.1436120)
Supplement: Supplementary file 1 [file DataSheet_1.pdf]

Supplementary Material

1 SUPPLEMENTARY DATA

1.1 Data preprocessing

After each data collection session, the individual point clouds from various scan locations were co-registered using SCENE software (FARO Technologies, Florida, US), version 2019.2. The raw point cloud data, stored in .FLS files, were imported and processed in SCENE. Only the *'Edge artifact'* filter was preselected for scan processing within SCENE. This filter allows for the identification and removal of noisy scan points at the edges of objects The *'Find Spheres'* checkbox was selected to automatically identify the registration spheres during processing. The *'Active Sphere Radii'* parameter was configured to 0.0994 meters.

To register the point clouds, the information gathered by the scanner's internal sensors, including color camera, inclinometer, compass, and GPS, were used within SCENE as initial scan placement. A subsequent automatic registration was performed using the *'Target Based* registration method based on the detected registration spheres. The quality of the registered point cloud was assessed using both target-based and mean point error statistics. Target-based statistics included the Euclidean distance between corresponding target pairs used for registration, while mean point error statistics computed the distance between local reference points and their corresponding references. Lower values for both metrics indicate a more accurate registration.

Once the spatial registration process was completed, the SCENE software's *'Clipping box'* tool was employed to isolate and extract the 3D points within the designated study area. The dimensions of the clipping boxes were manually adjusted to encompass the field boundaries defined by the scan locations at the outermost perimeter. Points within the clipping box were saved in the LASer (.LAS) file format, while all remaining points outside this region were excluded from the final point cloud dataset.

Co-registered point clouds were preprocessed to prepare them for subsequent analysis using the CloudCompare software, version 2.11.3 (Supplementary Figure S1). The preprocessing involved point cloud denoising and subsampling. For denoising, we applied a statistical outlier removal (SOR) filter with $N = 20$ and ± 2.5 standard deviations as outlier boundaries, eliminating points significantly distant from their neighbors. To further reduce point cloud size and manage computational demands while preserving spatial information, we introduced a subsampling step based on point-to-point distances. This step selectively retained points from the cloud using a distance threshold value, resulting in a new point cloud where any two points were at a distance equal to or greater than the specified value. We tested several distance threshold (i.e., 20 mm, 10 mm, 5 mm, and 1 mm), and found that a 5 mm threshold provided sufficient point cloud size reduction to enable posterior processing.

After subsampling, point cloud height was normalized with respect to the ground surface to correct for potential fluctuations in elevation within the field due to variations in terrain topography. This normalization process involved the creation of a digital terrain model (DTM) as a local reference (Supplementary Figure S1). First, the preprocessed point clouds were sampled using a grid with dimensions of 0.5 meters by 0.5 meters to obtain a rasterized sparse point cloud. Post-rasterization, any non-terrain points that may have been sampled would likely be spatially distant from their neighborhood points, facilitating their identification as outliers. Subsequently, a noise filter was applied to the sparse cloud to eliminate points

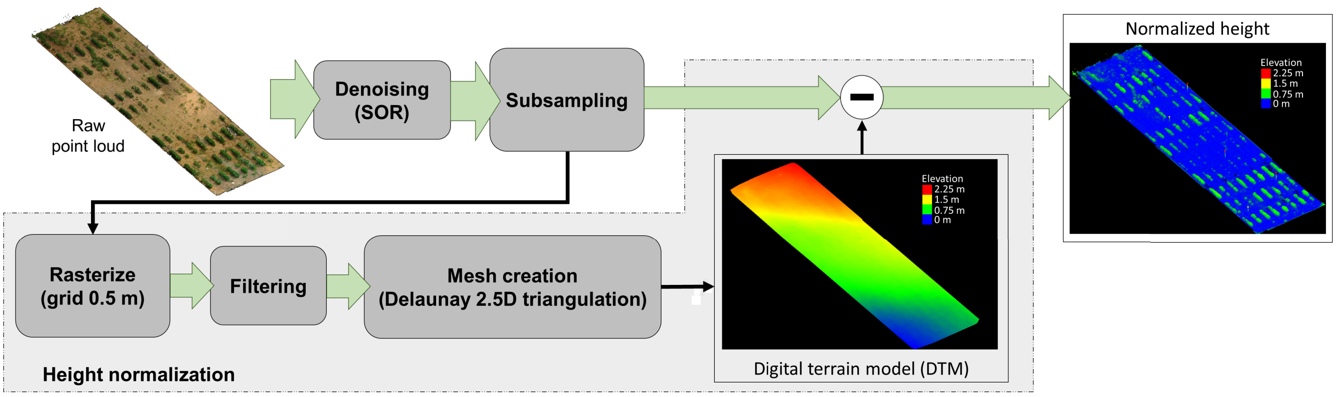

Supplementary Figure S1. Steps for point cloud data preprocessing after spatial registration.

that deviated significantly from the underlaying surface. Then, using the Delaunay triangulation method over the filtered sparse cloud, a mesh was reconstructed. This triangulation was executed on the local plane that best fitted the point cloud projection, leading to the creation of a DTM representing the field's surface. Finally, the height value of the DTM was subtracted from the elevation of each point in the point cloud, completing the height normalization procedure.

2 SUPPLEMENTARY TABLES AND FIGURES

2.1 Tables

Supplementary Table S1. Parametrization of common growth models of the Richards family curves for sigmoidal growth.

Model	T_i form
Logistic	$W = A\left(1 + e^{-k(t-T_i)}\right)^{-1}$
Gompertz	$W = Ae^{-e^{-k(t-T_i)}}$
3P-Richards	$W = A\left(1 + 3e^{-k(t-T_i)}\right)^{-\frac{1}{3}}$

Supplementary Table S2. Spatial registration statistics per data collection. DAP: Days after planting.

DAP	Registration errors (mm)		Original point clouds		Processed point clouds	
	Mean target error	Mean point error	#Points (M)	Size (GB)	#Points (M)	Size (GB)
35	0.9	5.0	110	2.8	6	0.24
42	1.7	3.8	106	2.7	15	0.38
49	0.9	4.6	161	4.1	16	0.42
56	1.3	4.2	224	5.7	18	0.46
62	1.3	5.1	288	7.3	17	0.44
70	2.1	6.9	361	9.1	23	0.59
77	2.2	6.4	353	8.7	25	0.65
84	2.3	8.5	348	8.8	25	0.62
98	3.2	7.9	332	8.1	23	0.58

Supplementary Table S3. Canopy height (CH) estimation results. Comparison of CH computed as the 95th percentile (CH95), the 99th percentile (CH99), and maximum height (CHmax). Subscript *u* indicates CH values obtained from non-temporal co-registered (unregistered) point clouds. Bold numbers indicate the best results.

Metric	CH95	$CH95_u$	CH99	$CH99_u$	CHmax	$CHmax_u$
R^2↑	0.919	0.904	0.941	0.929	**0.945**	0.930
RMSE (cm)↓	9.18	9.98	7.84	8.60	**7.56**	8.53
MAPE (%)↓	6.59	7.02	5.54	5.98	**5.34**	5.92

Supplementary Table S4. Comparison of growth models performance. Degrees of freedom (df), log-likelihood (logLik), Akaike information criterion (AIC), and Bayesian information criterion (BIC) values for each model and trait combination.

		CH			CV		
	df	ΔAIC↓	ΔBIC↓	logLik↑	ΔAIC↓	ΔBIC↓	logLik↑
**Logistic**	31	0	0	786.3	0	0	-273.0
Gompertz	31	54	54.1	759.3	10.3	10.3	-278.2
3P-Richards	31	4.1	4.2	784.2	38.7	38.7	-292.4

2.2 Figures

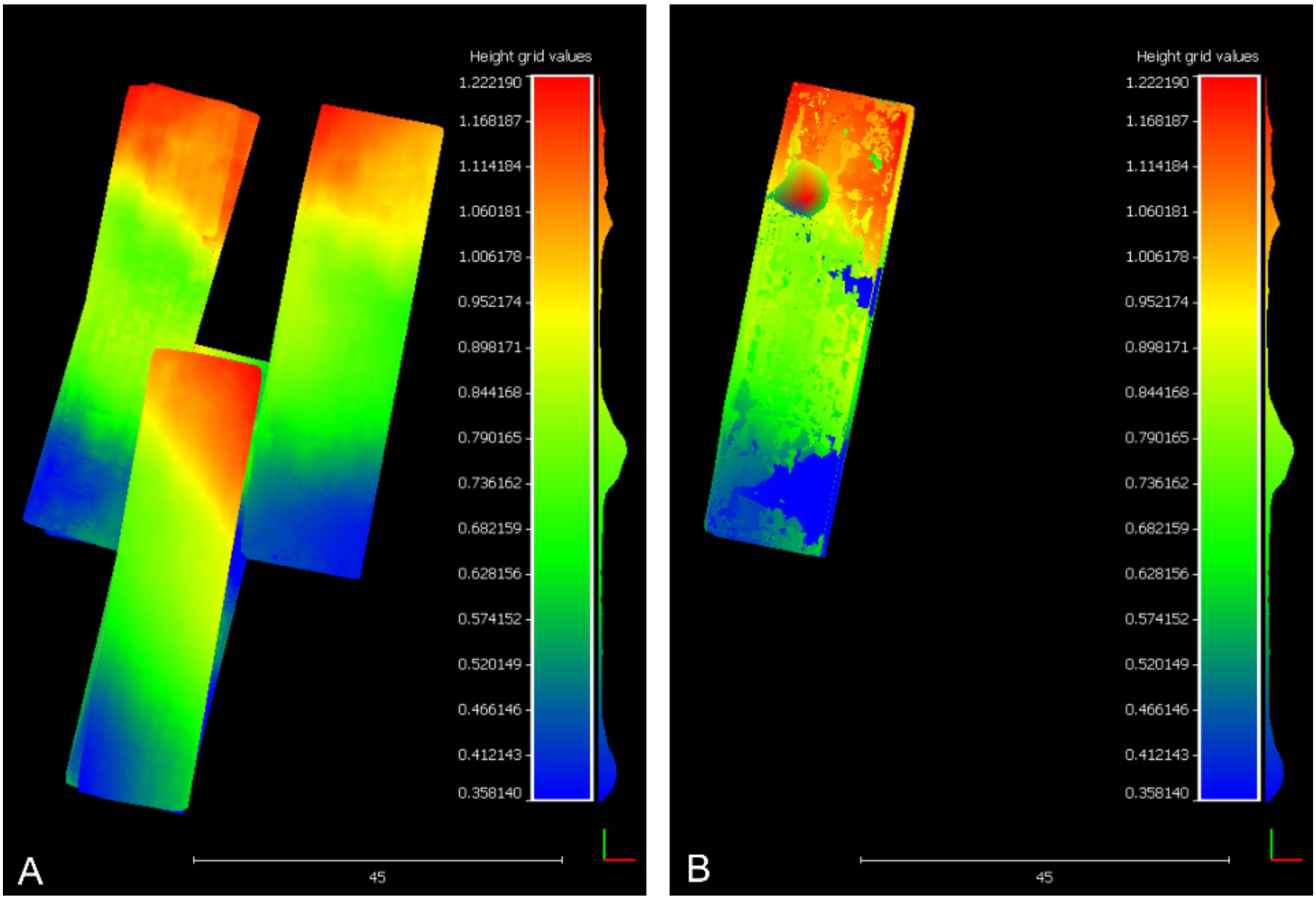

Supplementary Figure S2. Results of terrain-based alignment. **(A)** Initial positions of the DTMs viewed from a top-down perspective. **(B)** Final positions of the meshes after ICP-based registration

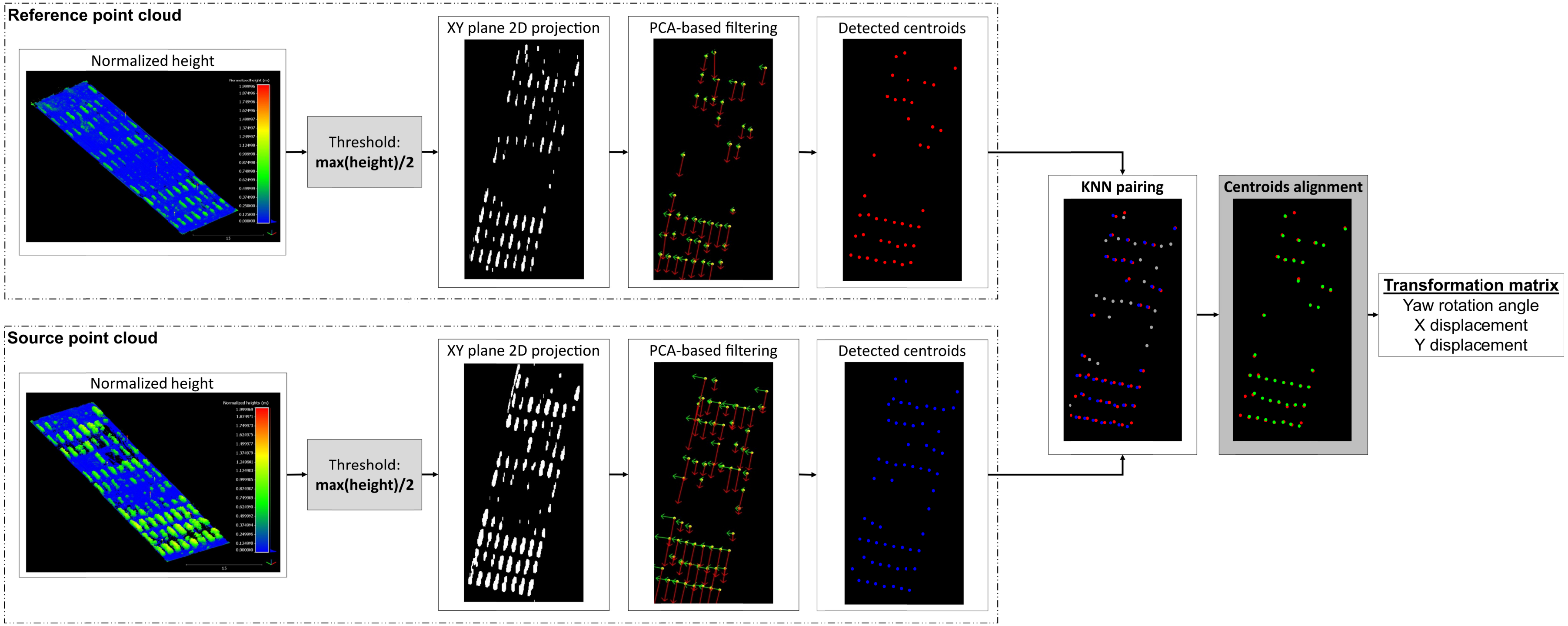

Supplementary Figure S3. Fine registration of two point clouds using bird's-eye view alignment. The reference point cloud data corresponds to the data collection session conducted at 35 DAP, while the source point cloud data corresponds to 62 DAP. Point clouds are colorized based on normalized height. In the 2D projection, white pixels represent the 3D vegetation points projected onto the XY plane. Red dots indicate plot centroids from the reference point cloud, while blue dots represent plot centroids from the source point cloud to be aligned. Following alignment, green dots denote the aligned plot centroids after minimizing pairwise neighbor distance errors.

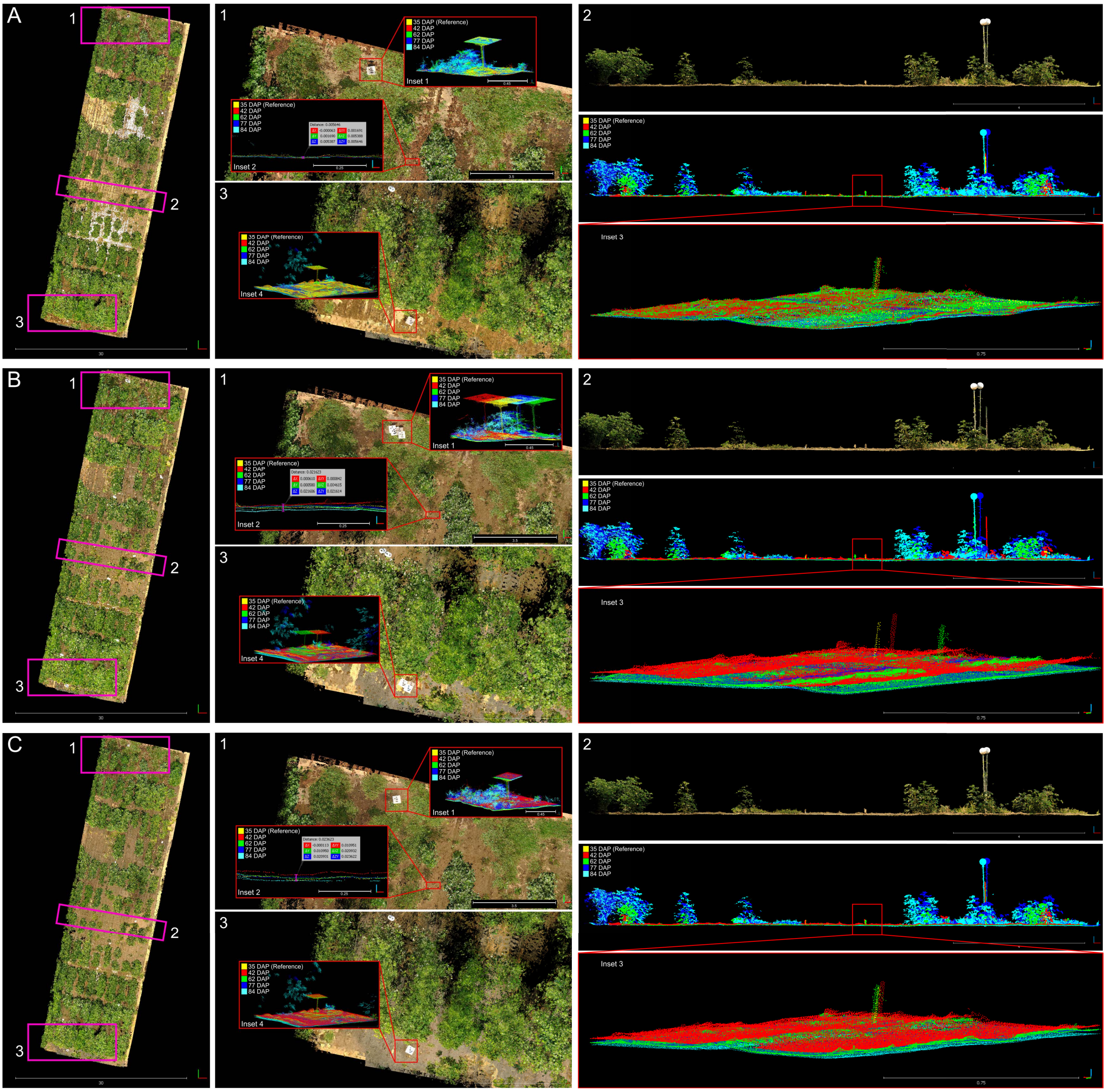

Supplementary Figure S4. Qualitative results comparison for registration of point clouds collected over time. **(A)** Manual alignment considered as the ground truth for performance evaluation. **(B)** Rough alignment results after the first alignment step based on digital terrain model matching. **(C)** Final alignment achieved after bird's-eye view refinement. Numbered windows indicate close-ups to the respective area of the field demarcated by a pink rectangle. Insets **(1)** demonstrate generalized alignment errors at the northern ground control point; Insets **(2)** highlight errors in the Z direction at the terrain level; Insets **(3)** show alignment errors in the center of the field; Insets **(4)** show generalized alignment errors at the southern ground control point. Different solid colors denote point clouds collected at different dates.

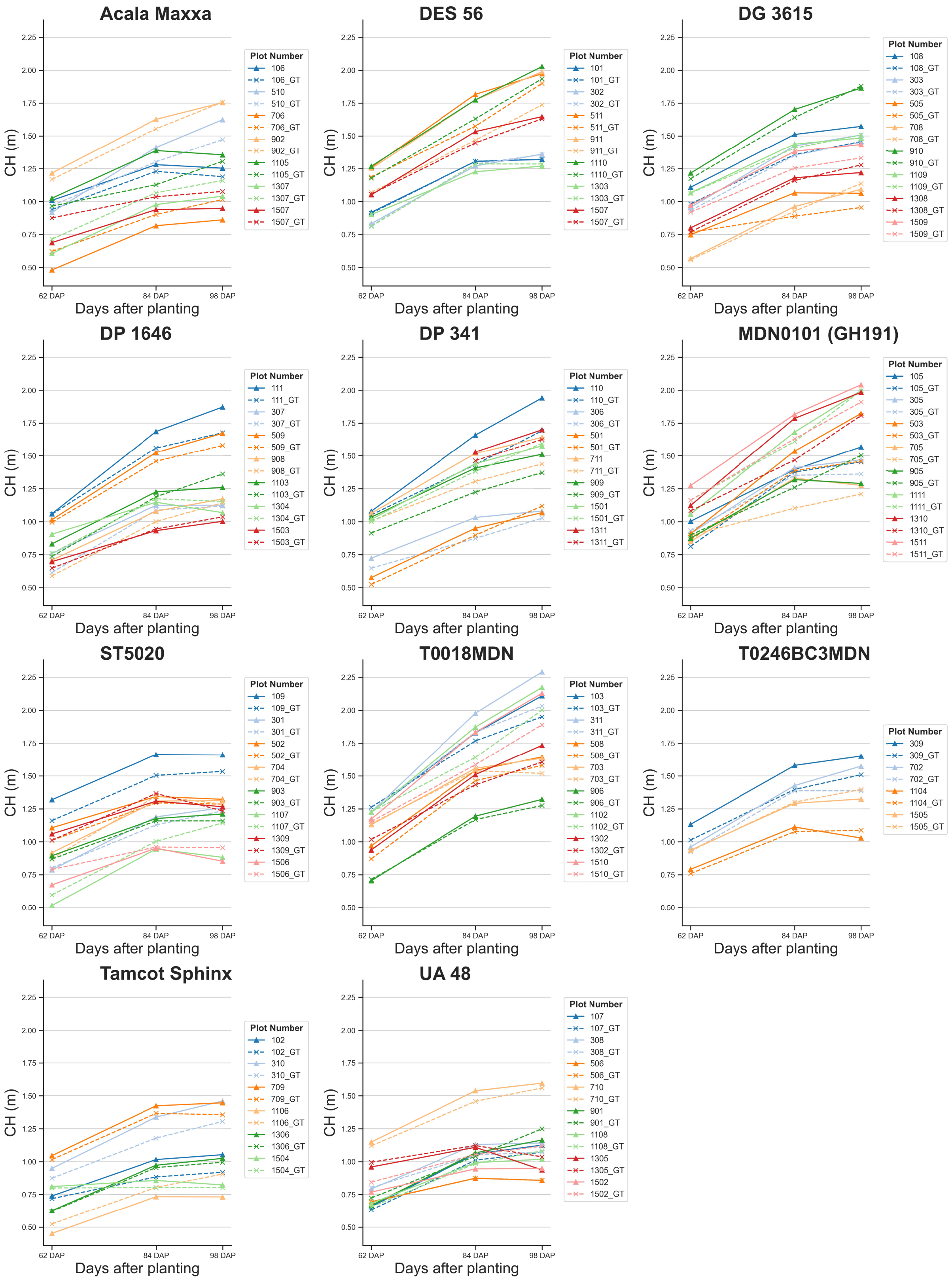

Supplementary Figure S5. Temporal variation of canopy height (CH) for individual plots within genotype. Temporal variation of predicted and observed CH per each plot. Solid lines represent CH values estimated using our methodology. Dashed lines represent ground truth (GT) CH values measured manually in the field. Each color and symbol combination represents a different genotype, with corresponding pairs.

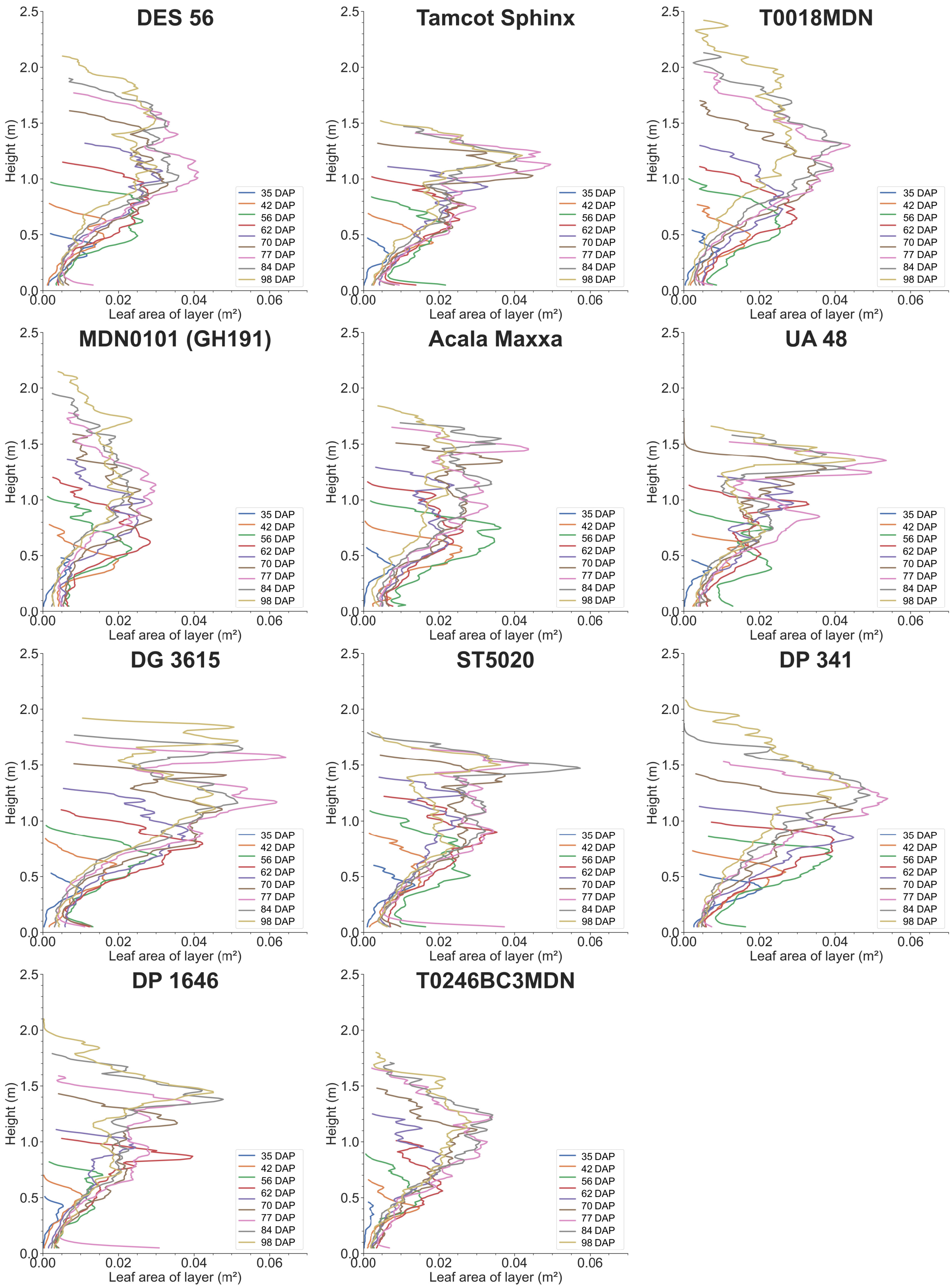

Supplementary Figure S6. Evolution of the vertical distribution of leaf area over time for 11 genotypes. Colored lines distinguish different data collection sessions. The TLS data have been binned into 1 cm height layers and smoothed to enhance visualization.
